# Drug-coated balloons versus drug-eluting stents in patients with in-stent restenosis: An updated meta-analysis with trial sequential analysis

**DOI:** 10.1186/s13019-024-03046-6

**Published:** 2024-11-06

**Authors:** Ahmed Abdelaziz, Karim Atta, Abdelrahman H. Hafez, Hanaa Elsayed, Ahmed A. Ibrahim, Mohamed Abdelaziz, Hallas Kadhim, Ahmed Mechi, Ahmed Elaraby, Mahmoud Ezzat, Ahmed Fadel, Abdullah Nouh, Rahma AbdElfattah Ibrahim, Mohamed Hatem Ellabban, Ali Bakr, Ahmed Nasr, Mustafa Suppah

**Affiliations:** 1 Medical Research group of Egypt (MRGE), Cairo, Egypt; 2 Faculty of Medicine, Al-Azhar University, Cairo, Egypt; 3 Institute of Medicine, National Research Mordovia State University, Saransk, Russia; 4 Faculty of Medicine, Zagazig University, Zagazig, Egypt; 5 Faculty of Medicine, Menoufia University, Menoufia, Egypt; 6 Al Muthanna University College of Medicine, Samawah, Iraq; 7 Internal Medicine Department, University of Kufa, Medicine College, Najaf, Iraq; 8 Faculty of Medicine, Sohag University, Sohag, Egypt; 9 Faculty of Medicine, Kafr Elsheikh University, Kafr Elsheikh, Egypt; 10 Faculty of Medicine New Damietta, Al-Azhar University, New Damietta, Egypt; 11 Department of Cardiovascular Medicine, Mayo Clinic, 13400 E Shea Boulevard, Scottsdale, AZ, 85259 USA

**Keywords:** DCB, DES, In-stent restenosis, PCI, Meta-analysis

## Abstract

**Background:**

Drug-coated balloons (DCB) have promising results in the management of in-stent restenosis (ISR), still their role remains a major challenge, and not well established in contemporary clinical practice.

**Aims:**

To provide a comprehensive appraisal of the efficacy and safety of DCBs in patients with in-stent restenosis (ISR).

**Methods:**

We searched PubMed, Scopus, web of Science, Ovid, and Cochrane Central from inception until 30 March, 2023. We included randomized controlled trials (RCTs) that compared DCB versus DES in ISR patients. Our primary endpoints were major adverse cardiac events (MACE) and late lumen loss (LLL). Secondary clinical endpoints were all-cause death, cardiac death, MI, TLR, TVR, and stent thrombosis, and angiographic outcomes were MLD, and in-stent binary restenosis.

**Results:**

Ten RCTs comprising 1977 patients were included in this meta-analysis. The incidence of MACE was 15.57% in the DCB group compared to 14.13% in the DES group, with no significant difference in the risk of MACE following DCB (odds ratio [OR] 1.04, 95% confidence interval [CI]: 0.87 to 1.44). Compared with the DES intervention, the risk of LLL was comparable to the DCB intervention (mean difference [MD] -0.08, 95% CI: -0.18 to 0.02), while the incidence of TLR was increased in the DCB intervention (OR: 1.54, 95% CI: 1.2 to 1.99).

**Conclusion:**

DCB was comparable to DES implantation is ISR patients regarding clinical outcomes, however it showed an increase in TLR events. Moreover, a RCT with large sample size and longer follow-up duration is warrened to validate these results.

**Supplementary Information:**

The online version contains supplementary material available at 10.1186/s13019-024-03046-6.

## Introduction

In-stent re-stenosis (ISR) commonly occurs in clinical settings subsequent to the implantation of bare metal stents (BMS) due to the augmented neointimal proliferation induced by these devices. Furthermore, drug-eluting stents (DES) may also exhibit the occurrence of ISR, particularly when implemented in unfavorable clinical circumstances and anatomical conditions [1–3]. The fundamental aim of percutaneous coronary intervention (PCI) is to optimize hemodynamic perfusion through the amelioration of narrowed coronary arteries. Nevertheless, the clinical efficacy of this minimally invasive technique has encountered prolonged impediments in the form of re-stenosis, an occurrence defined by the subsequent re-narrowing of previously treated arteries, which has persisted since its inception in 1977 [4].

Compared to bare-metal stents (BMS) and conventional balloon angioplasty (BA), DES has demonstrated a substantial decrease in re-stenosis likelihood [5–7]. Nevertheless, because of the ongoing rise in DES implantation, the rate of DES re-stenosis is still significant [8]. Despite the widespread use of new-generation DES, DES-ISR continues to occur in 5–10% of patients after DES deployment and has established itself as a common clinical problem [9, 10].

Drug-coated balloon (DCB) is a novel therapeutic approach for BMS-ISR and DES-ISR; studies have shown that it is associated with better outcomes when compared to other traditional treatment techniques [9]. Previous randomized studies have examined the application of DCB in patient populations exclusively comprised of either BMS-ISR or DES-ISR individuals. However, no study has included patients with any form of in-stent re-stenosis (i.e., both BMS-ISR and DES-ISR [4].

Notably, recent clinical practice guidelines imply that these 2 therapy methods (DES and DES) presently constitute the best treatments available (both with the level of recommendation IA) for patients with ISR DCB are at least as effective as first-generation DES in these patients as first-generation DES and are more effective than conventional therapy approaches [1, 11]. The comparative efficacy of DCB versus “new generation” DES in patients with ISR is largely unestablished.

DCB is an attractive strategy for managing recurrent DES-ISR due to its potential to obviate the need for additional metallic layers and prolonged dual anti-platelet treatment (DAPT). Nonetheless, there is a scarcity of research investigating the utilization of DCB for the treatment of recurrent DES-ISR. The outcomes obtained thus far are subject to debate [9]. The objective of our study was to conduct a comparative analysis of the efficacy and safety of DCB and DES in the management of ISR.

## Methods

In this meta-analysis, we adhered to the guidelines outlined in the Preferred Reporting Items for Systematic Reviews and Meta-Analysis (PRISMA) statement, which outlines guidelines for transparently reporting systematic reviews and meta-analyses. Additionally, we adhered to the guidelines outlined in the Cochrane Handbook of Systematic Reviews and Meta-Analysis of Interventions (version 5.1.0) [12].

### Eligibility criteria

In our analysis, we incorporated all randomized controlled trials that focused on patients with in-stent re-stenosis undergoing PCI and treated with drug-coated balloons as the intervention group and drug-eluting stents as the control group. Our study reported outcomes of interest using intention-to-treat analysis and excluded animal studies, conference abstracts, and unpublished data. We did not limit our search to English language studies and considered foreign language studies as part of our inclusion criteria.

### Primary and secondary outcomes

Our primary investigation focused on evaluating the incidence of Major Adverse Cardiovascular Events (MACE) and late lumen loss (LLL), utilizing the definitions provided by each respective author. Additionally, we conducted an analysis of several secondary outcomes of interest, including all-cause death, cardiac death, myocardial infarction (MI), target lesion revascularization (TLR), target vessel revascularization (TVR), in-segment binary re-stenosis, stent thrombosis, and minimum lumen diameter (MLD). The definitions for these outcomes were based on the definitions explicitly reported by the authors of the individual studies incorporated in our analysis.

### Literature search

A comprehensive electronic search was conducted across multiple databases including PubMed, Scopus, Web of Science, Ovid, and the Cochrane central library. The search encompassed articles published from the inception of these databases up until March 30th, 2023. The search strategy employed in this study was as follows:

Scopus: (“drug-coated balloon” OR “DESs”) AND (“Drug-Eluting Stent” OR “DES” OR “PCI”) AND (“in-stent re-stenosis” OR “re-stenosis” OR “coronary Stent re-stenosis”) AND (“coronary artery disease*” OR “myocardial infarction”) AND (“PCI” OR “Percutaneous Coronary Intervention”).

Others: (drug-coated balloon OR DESs) AND (Drug-Eluting Stent OR DES OR PCI) AND (in-stent re-stenosis OR re-stenosis OR coronary Stent re-stenosis) AND (coronary artery disease* OR myocardial infarction) AND (PCI OR Percutaneous Coronary Intervention).

The duplicates were identified and removed using EndNote. We screened all relevant included studies and prior meta-analyses for additional citations.

### Screening of the literature search results

The results were screened in a two-step way; the first was a title and abstract screening, then a full-text screening of the relevant studies.

### Data extraction

A designated data extraction sheet was employed for the purpose of acquiring essential information. The data extracted encompassed four primary domains, namely: (1) the distinctive attributes of the studies incorporated in the analysis, (2) the characteristics of the population under study, and (3) the domains associated with the evaluation of potential bias, and (4) Outcome measures, which included MACE, all-cause death, cardiac death, MI, TLR, TVR, in-segment binary re-stenosis, stent thrombosis, LLL, and MLD. Data was extracted independently, in duplicate, compared for discrepancies, and that any disputes were resolved by consensus.

### Synthesis of results

We pooled the reported outcomes based on the follow-up duration, in which short-term follow-up was defined as a follow-up less than or equal to 1 year, while long-term follow-up was defined as a follow-up longer than 1 year. In cases where studies provided data of long-term follow-up at multiple time points, we focused on the outcomes reported at the final follow-up point. We used the DerSimonian-Laird random-effect model to pool the frequency of events and the total number of patients for dichotomous outcomes, reporting the results as odds ratios (OR). We used the DerSimonian-Laird random-effect model for continuous outcomes to pool the mean difference (MD) and its 95% confidence interval (CI). We used Stata MP Version 17 for Mac to conduct all analyses.

### Assessment of heterogeneity

The Chi-square test (specifically the Cochrane Q test) was utilized to assess the statistical heterogeneity across the studies. The evaluation was conducted by employing the following equation: $\left( {\frac{{Q - df}}{Q}} \right)x100\%$ A P-value of less than 0.1 for the Chi-square test was considered significant heterogeneity. We defined high heterogeneity as I-square values of 50% or greater. In cases of significant heterogeneity, we utilized the leave-one-out sensitivity analysis model to address the reported heterogeneity. Additionally, we used the Galbraith plot to detect any heterogeneity across studies.

### Quality assessment

We evaluated the quality of the clinical trials included in our analysis using the Cochrane Risk of Bias 2 (ROB-2) tool for randomized controlled trials (RCTs). This tool assesses the risk of bias in five domains: selection bias, performance bias, detection bias, attrition bias, and reporting bias [13]. The authors’ determinations were categorized into three levels of bias assessment: ‘High risk of bias’, ‘Some concerns’, or ‘Low risk of bias’. To investigate the potential existence of publication bias across the included studies, we employed the funnel plot model to assess the relationship between effect size and standard error. In order to evaluate the presence of publication bias more comprehensively, we conducted Egger’s regression test.

Due to the limited amount of data reported and the cumulative pooling of trials, there is an increased risk of type 1 and type 2 errors. To address this, we used Trial Sequential Analysis (TSA) to determine if the evidence from the pooled trials is conclusive and reliable. The intervention’s confidence level is considered conclusive and sufficient when the z-line on the curve crosses both the conventional boundary and the boundary of sequence monitoring, indicating that no further studies are necessary. Conversely, if the z-line on the curve does not cross any boundary, the evidence is insufficient, and further studies are necessary. In this meta-analysis, we used an alpha error of 0.05 and a beta error of 80% power. We calculated the mean difference in the meta-analysis to determine the sample size required for TSA.

## Results

### Literature search results

**Fig. 1:**
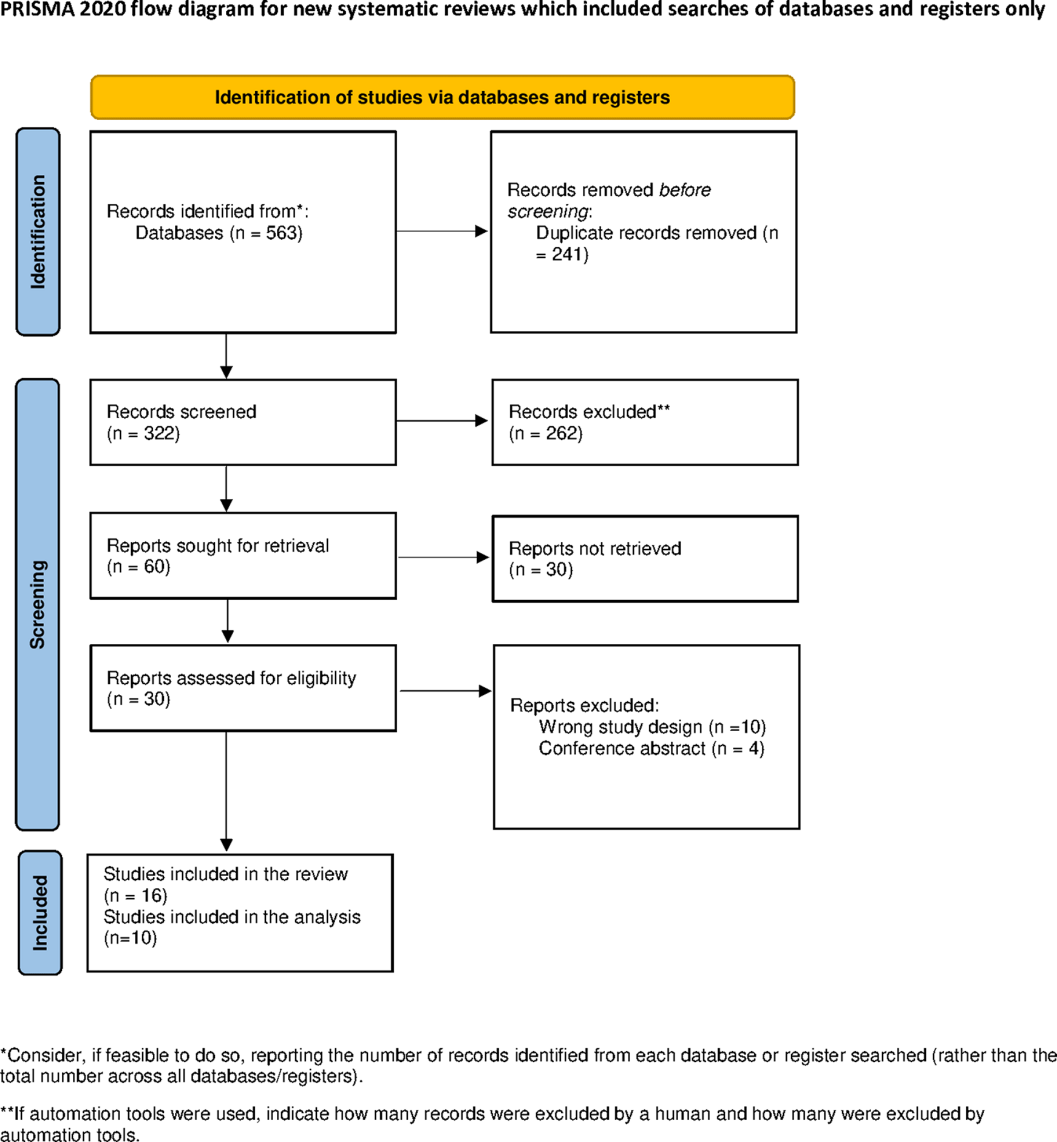
PRISMA flow chart of our included studies

A total of 563 articles were retrieved through a comprehensive electronic search. After title and abstract screening, 60 studies were eligible, and following full-text screening, 10 studies and further 6 follow-up studies were included in this systematic review and meta-analysis. The PRISMA flow chart is shown in Fig. 1.

### Included studies characteristics

Our meta-analysis included 10 studies [3, 4, 6, 10, 11, 14–18]. with 6 follow-up extension studies [1, 7, 19–22]. comprising 1977 patients comparing DCB with the DES intervention. All studies assessed our primary clinical outcome, MACE, and our angiographic outcome, LLL. Baseline characteristics and a summary of included studies are shown in Table 1.

**Table 1:** Baseline and summary characteristics of included studies

Characteristics of All included Studies																							
Author, year	Country	Patients (n)	Age (mean)	male, n	angio follow up (months)	clinical follow up (months)	DCB type	DES type	RVD, mean	MLD, mean	Medical Conditions, n						Target vessel, n			DS, mean	ISR Mehran classification, n		
											History of CABG	History of MI	DM	HTN	Hyperlipidaemia	Current smoker	LAD	LCX	RCA		I	II	III-IV
Pleva 2016	Czechia	68/68	65.6/65.5	43/46	12	12	Paclitaxel-Eluting Balloon	Everolimus-Eluting Stents	2.64/2.66	0.92/0.79	3\6	43/41	17/18	NA	NA	31/29	35/40	NA	22/22	71.8/78.0	30/21	34/35	10\18
Pleva 2018 (follow-up)	Czechia	68/68	65.6/65.5	43/46	NA	36	Paclitaxel-Eluting Balloon	Everolimus-Eluting Stents	2.64/2.66	0.92/0.79	3\6	43/41	17/18	NA	NA	31/29	35/40	NA	22/22	71.8/78.0	30/21	34/35	10\18
Unverdorben 2009	Germany	66/65	64.6/ 65.1	48/50	6	12	Paclitaxel-Coated Balloon	paclitaxel-eluting stent	2.85/2.83	0.74 / 0.77	NA	NA	22/17	53/54	52/46	16/15	20/28	24/19	22/17	73.9/72.8	31/25	20/26	15/14
Unverdorben 2015 (follow up)	Germany	66/65	64.6/ 65.1	48/50	6	36	Paclitaxel-coated balloon	paclitaxel-coated stent	2.85/2.83	0.74/ 0.77	NA	NA	22/17	53/54	52/46	16/15	20/28	24/19	22/17	73.9/72.8	31/25	20/26	15/14
Wong 2018	China	86/86	67/ 66	61/62	14	12	Paclitaxel-Coated Balloon	everolimus-eluting stents	2.85/ 3.06	0.63/ 0.63	NA	26/22	43/38	60/65	49/53	40/37	48/52	13\11	24/21	77\79	45/45	1\10	12-Oct
Xu 2014	China	109/106	61.8/ 62.1	88/86	9	12	Paclitaxel-Coated Balloon	Paclitaxel-Eluting Stent	2.66/ 2.72	2.63/ 2.45	3/0	53/37	44/35	78/69	38/35	23/27	47/61	21/13	45/34	68.26/68.43	67\54	21/22	15/23
Xu 2016 (follow-up)	China	109/106	61.8/ 62.1	88/86	NA	24	Paclitaxel-Coated Balloon	Paclitaxel-Eluting Stent	2.66/ 2.72	2.63/ 2.45	3/0	53/37	44/35	78/69	38/35	23/27	47/61	21/13	45/34	68.26/68.43	67\54	21/22	15/23
Adriaenssens 2014	Belgium	25/25	67/64	18/25	9	12	Paclitaxel-Coated Balloon	everolimus-eluting stent	3/2.847	0.98/0.57	NA	12\10	6\1	16/15	24/24	5\3	6\11	5\7	13\6	67.7/79.4	8\9	13\10	4\6
Alfonso 2014	Spain	95/94	67/64	82/82	9	12	Paclitaxel-Coated Balloon	everolimus-eluting stent	2.64/2.64	1.02/0.93	4\7	57/56	30/19	68/68	69/62	NA	35/37	21/22	37/32	61/65	38/34	45/42	12\18
Alfonso 2015	Spain	154/155	66/66	127/130	9	12	Paclitaxel-Coated Balloon	everolimus-eluting stents	2.59/2.67	0.79/0.75	16/17	73/77	75/66	110/121	110/121	NA	77/71	27/34	43/45	69/72	97/99	53/44	4\12
Alfonso 2016 (follow-up)	Spain	95/94	67/64	82/82	NA	36	NA	Everolimus-Eluting Stent	2.64/2.64	1.02/0.93	NA	NA	30/19	68/68	69/62	NA	35/37	21/22	37/32	61/65	38/34	45/42	12\18
Alfonso 2018 (follow-up)	Spain	154/155	66/66	127/130	NA	36	NA	Everolimus-Eluting Stent	2.59/2.67	0.79/0.75	NA	NA	75/66	110/121	110/121	NA	77/71	27/34	43/45	69/72	97/99	53/44	4\12
Baan 2018	Netherlands	137/141	66/65	72/84	6	12	Paclitaxel-Coated Balloon	everolimus-eluting stents	2.56/2.59	0.77/0.79	14/16	53/52	31/33	64/67	59/60	17/13	41/39	20/24	37/35	69.7/69.3	51/53	32/34	NA
Byrne 2013	Germany	137/131	67.7/68.8	105/88	8	12	Paclitaxel-Coated Balloon	paclitaxel-eluting stents	NA	0·97/0·93	15/32	53/50	56/61	105/101	108/103	19/15	59/50	54/61	59/56	64.4/66.7	119/110	44/49	9\9
Kufner 2015 (follow-up)	Germany	137/131	67.7/68.8	105/88	NA	36	Paclitaxel-Coated Balloon	paclitaxel-eluting stents	NA	0·97/0·93	15/32	53/50	56/61	105/101	108/103	19/15	59/50	54/61	59/56	64.4/66.7	119/110	44/49	9\9
Jensen 2018	Germany	157/72	67.2/69.4	122/49	6	18	Paclitaxel-Coated Balloon	sirolimus-eluting stent (SES)	3/2.9	1/0.9	NA	93/35	48/24	144/70	134/62	NA	NA	NA	NA	67.2/68.9	105\53	46/17	NA

The data are presented as DCB/DES. Angio: Angiographic; DCB: drug-coated balloons; DES: drug-eluting stents; RVD: reference vessel diameter; MLD: mean lumen diameter; DS: diameter stenosis; ISR: in-stent restenosis

### Risk of bias assessment

**Fig. 2:**
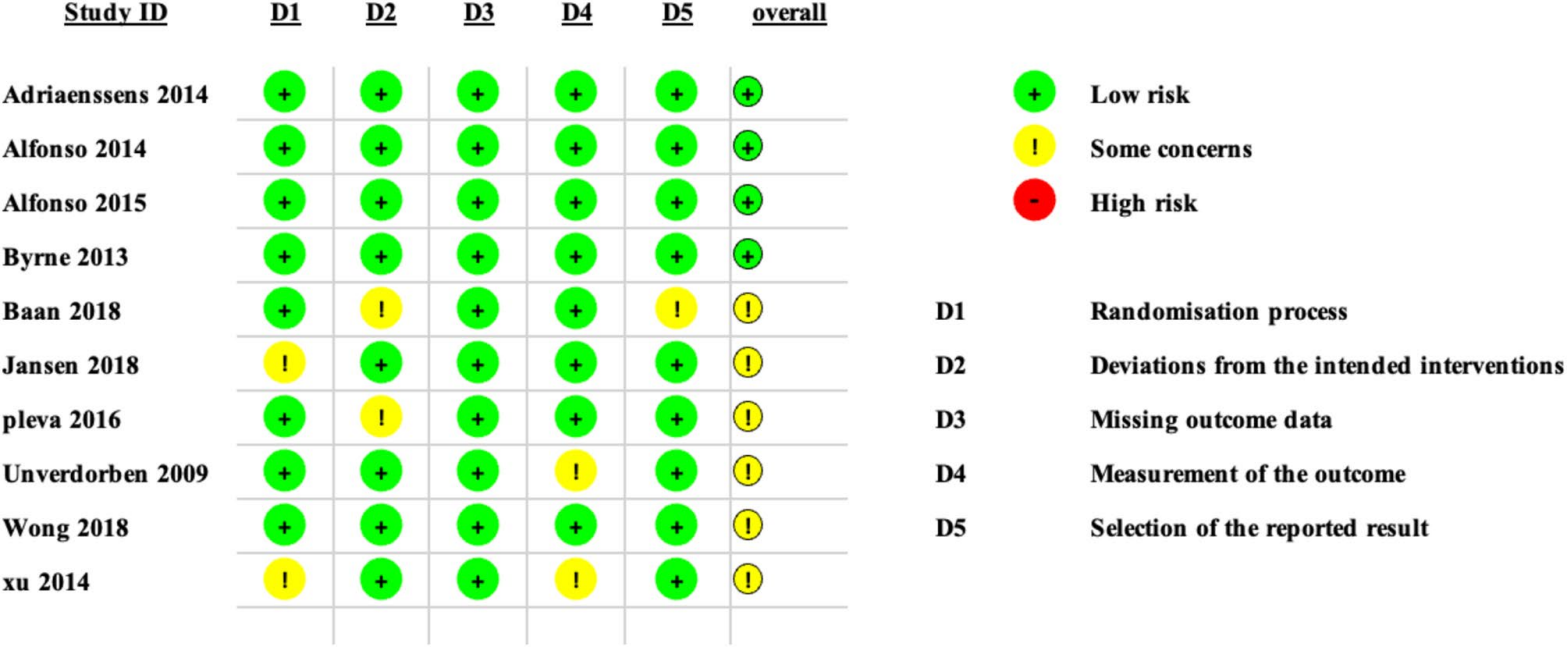
Risk of bias assessment tool-2 (ROB-2)

A summary and graph of the risk of bias assessment of our included studies are shown in Fig. 2. Most studies showed an overall unclear bias; however, seven studies showed an overall low risk of bias. The authors’ judgement was made according to the Cochrane risk of bias assessment tool.

#### MACE

**Fig. 3:**
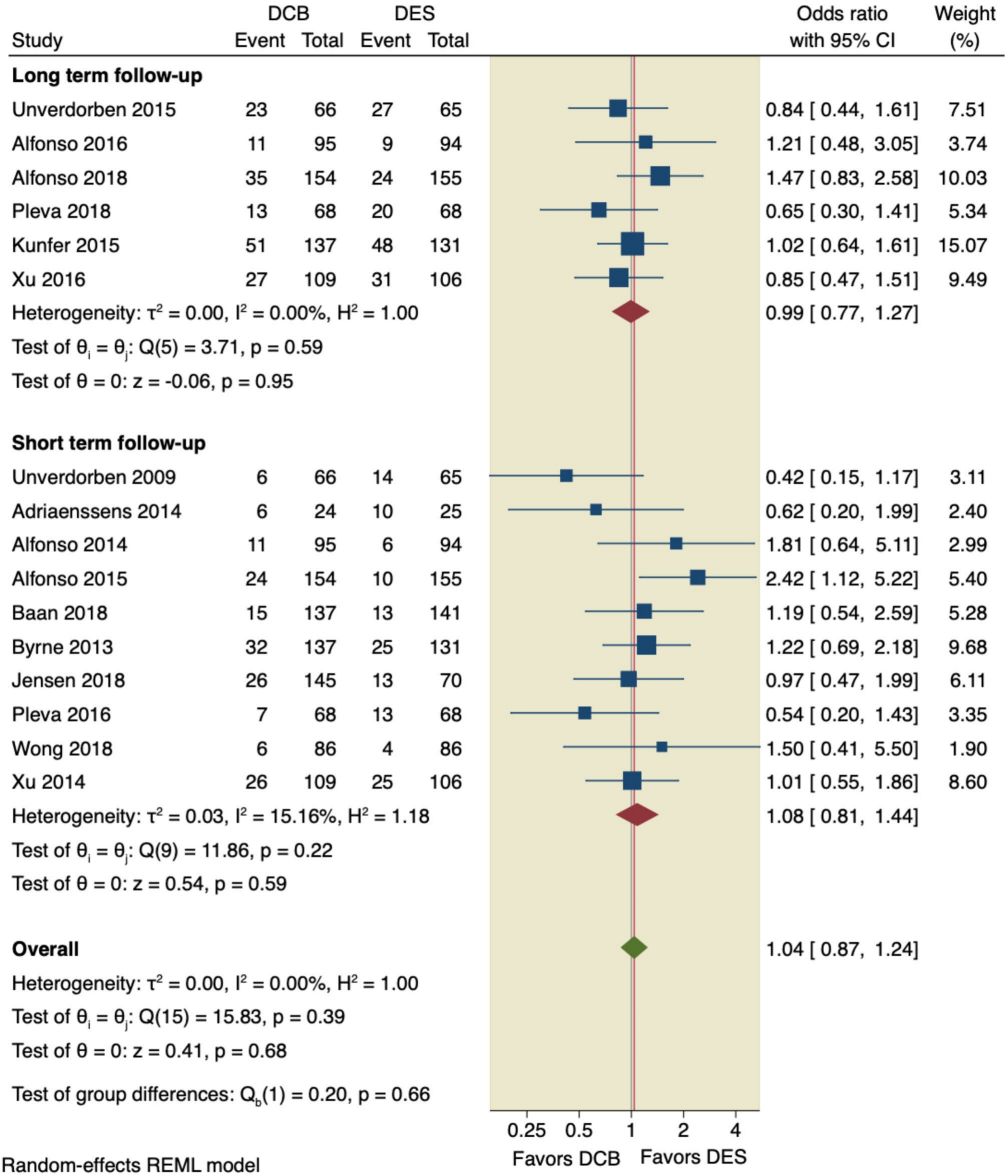
Pooled estimates from RCTs evaluating the effect of DCB on the incidence of MACE with a random-effects model. DCB: Drug-coated balloons; DES: drug-eluting stents; CI: confidence interval

All included studies assessed MACE with an overall incidence rate of 15.57% (159 of 1021) in the DCB group compared to 14.13% (133 of 941) in the DES group. We ran the analysis in two scenarios; the pooled OR based on the follow-up duration showed no significant difference between the DCB intervention and the DES intervention to reduce the incidence of MACE (OR = 1.04, 95% CI [0.87, 1.24], *p* = 0.68). Subgroup analysis revealed no significant difference between the two studies interventions. On the other hand, the pooled analysis of 6 studies that assessed long-term incidence revealed no significant difference between the two interventions used (OR = 0.99, 95% CI [0.77, 1.27], *p* = 0.95). The pooled studies were not heterogenous (I^2^ = 0.00%, *p* = 0.39), as shown in Fig. 3.

**Fig. 4:**
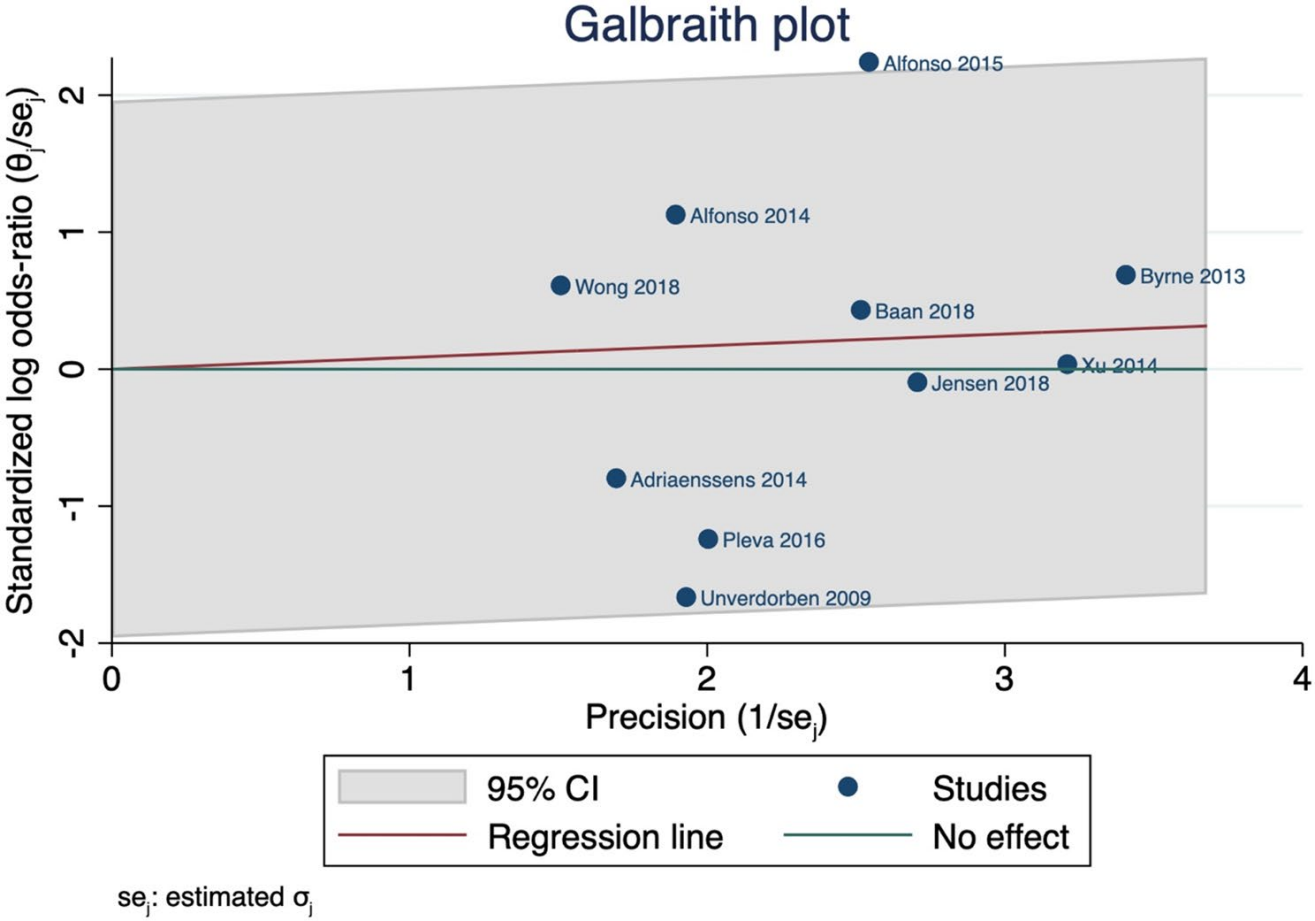
Galbraith plot indicating the heterogeneity across studies assessing MACE. CI: confidence interval

We also used the Galbraith plot to detect the heterogeneity, and by inspection, only one study was considered outliers, indicating its heterogeneity from other studies, as shown in Fig. 4. Insufficient literature search and clinical heterogeneity may attribute to the current evidence.

**Fig. 5:**
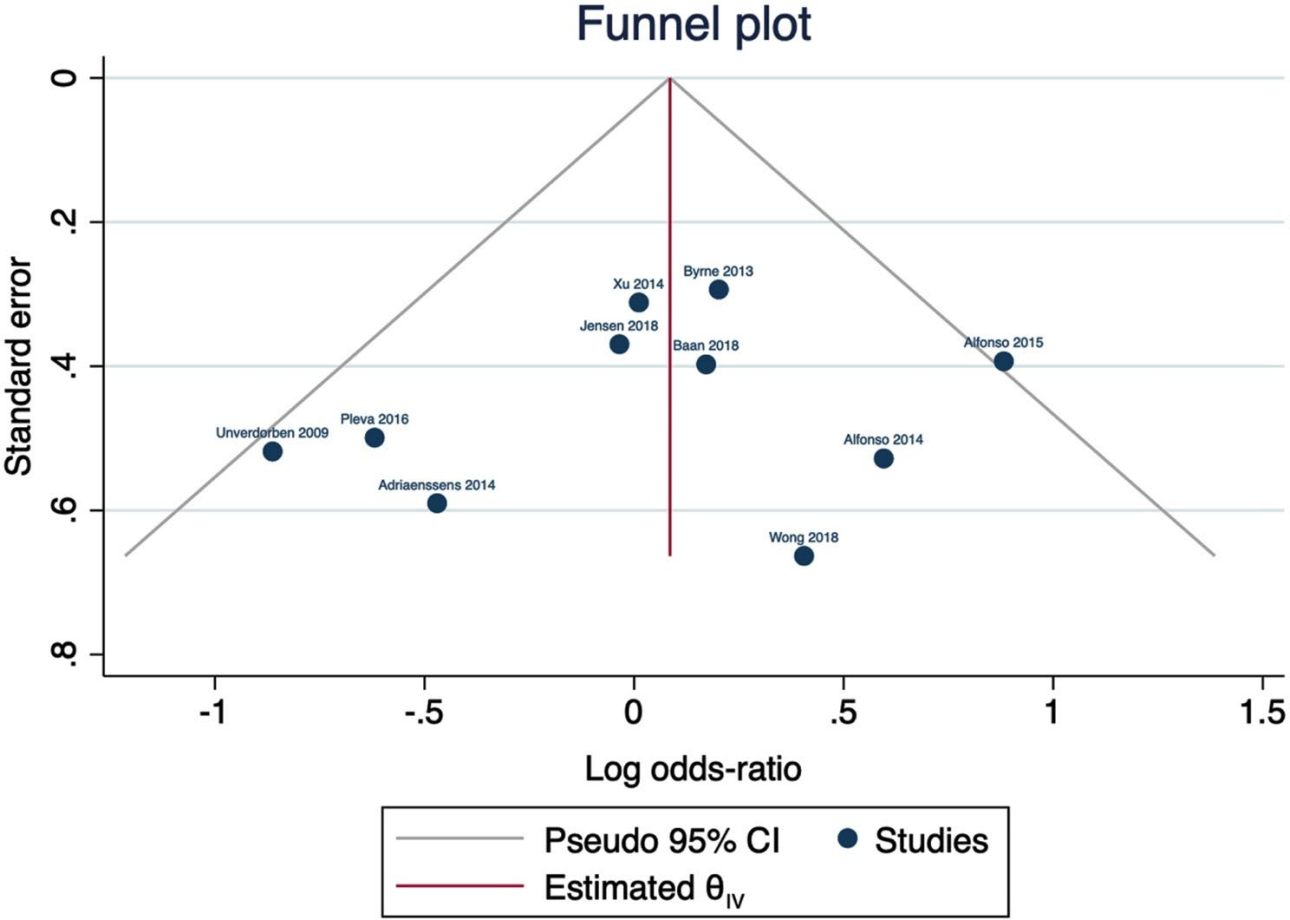
Funnel plot for possible publication bias regarding MACE

We used the funnel plot to detect possible publication bias, and by inspection, only one study was located outside the 95% CI of precision area, indicating that there was a possible publication bias; however, when we used the trim and fill method, no studies were imputed assuming possible stability across studies, as shown in Fig. 5.

We performed another analysis based on the type of ISR studies, and the pooled analysis showed that DCB was comparable to DES in either BMS ISR or DES ISR with the following values (OR: 0.78, 95% CI: 0.56 to 1.09, *p* = 0.15 in BMS ISR, and 1.16, 95% CI: 0.94 to 1.44, *p* = 0.16 in DES ISR), as shown in Supplementary Fig. S1.

**Fig. 6:**
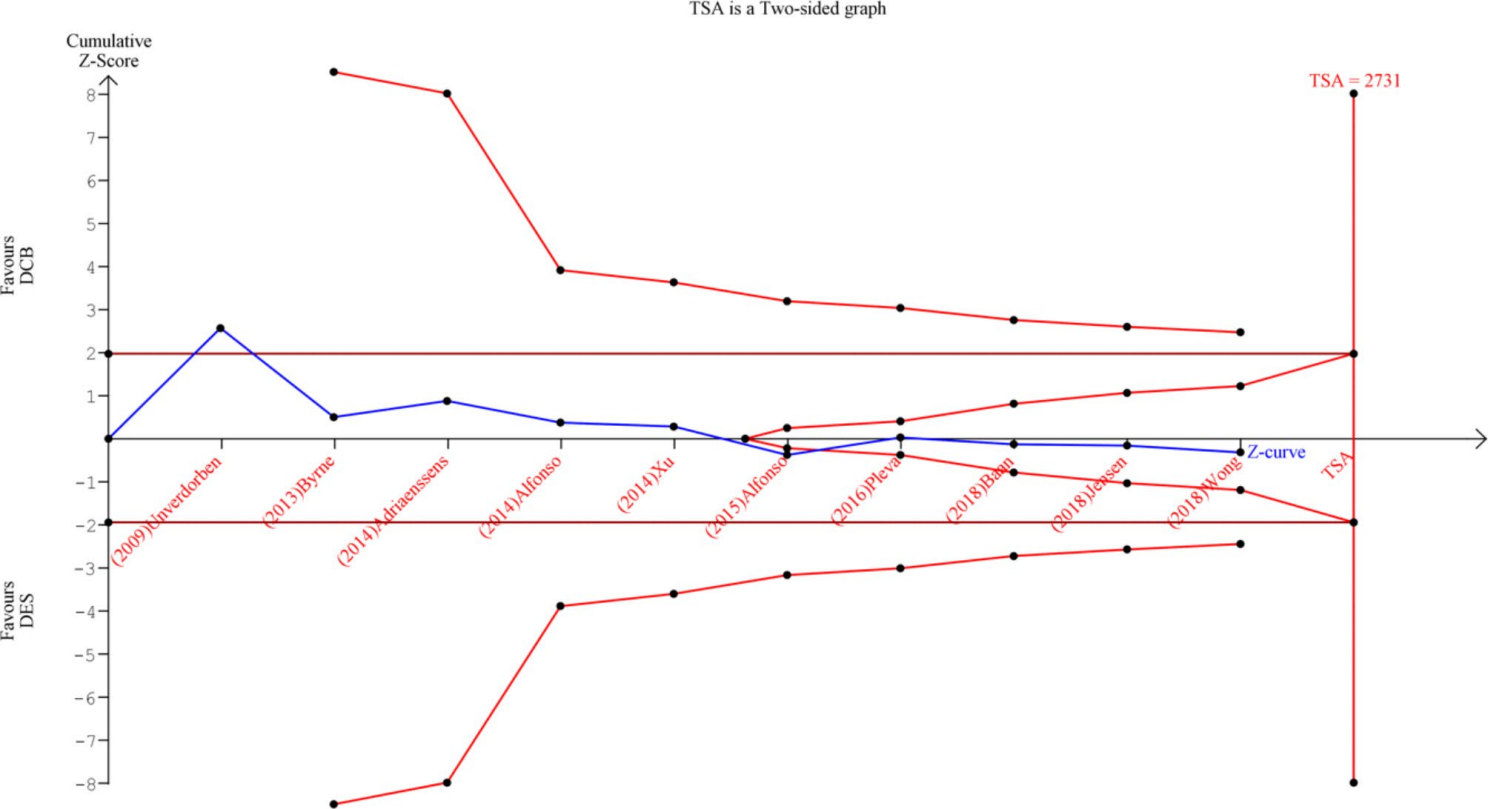
A trial sequential analysis (TSA) for 10 RCTs illustrating that the cumulative Z-curve crossed the conventional boundary for benefit only and did not cross the trial sequential monitoring boundary for benefit, establishing sufficient but not conclusive evidence and suggesting further trials are still needed to validate our results. A diversity-adjusted required information size of 2731 patients was calculated using an alpha error of 0.05, a beta error of 0.20 (power 80%), an anticipated RR reduction of 10% in DCB, and a control event proportion of 14.13%, as calculated from the control group in this meta-analysis. RCT: randomised controlled trial; RR: risk ratio

We performed a trial sequential analysis (TSA) on 10 studies that assessed MACE excluding studies of follow-up of which the cumulative Z-line on the curve crossed the conventional boundary of benefit only; and did not cross the trial sequential monitoring boundary, suggesting that DCB is comparable to DES to decrease the incidence of MACE, and further large volume RCTs should be carried out to validate our results, as shown in Fig. 6.

#### Late lumen loss (LLL)

**Fig. 7:**
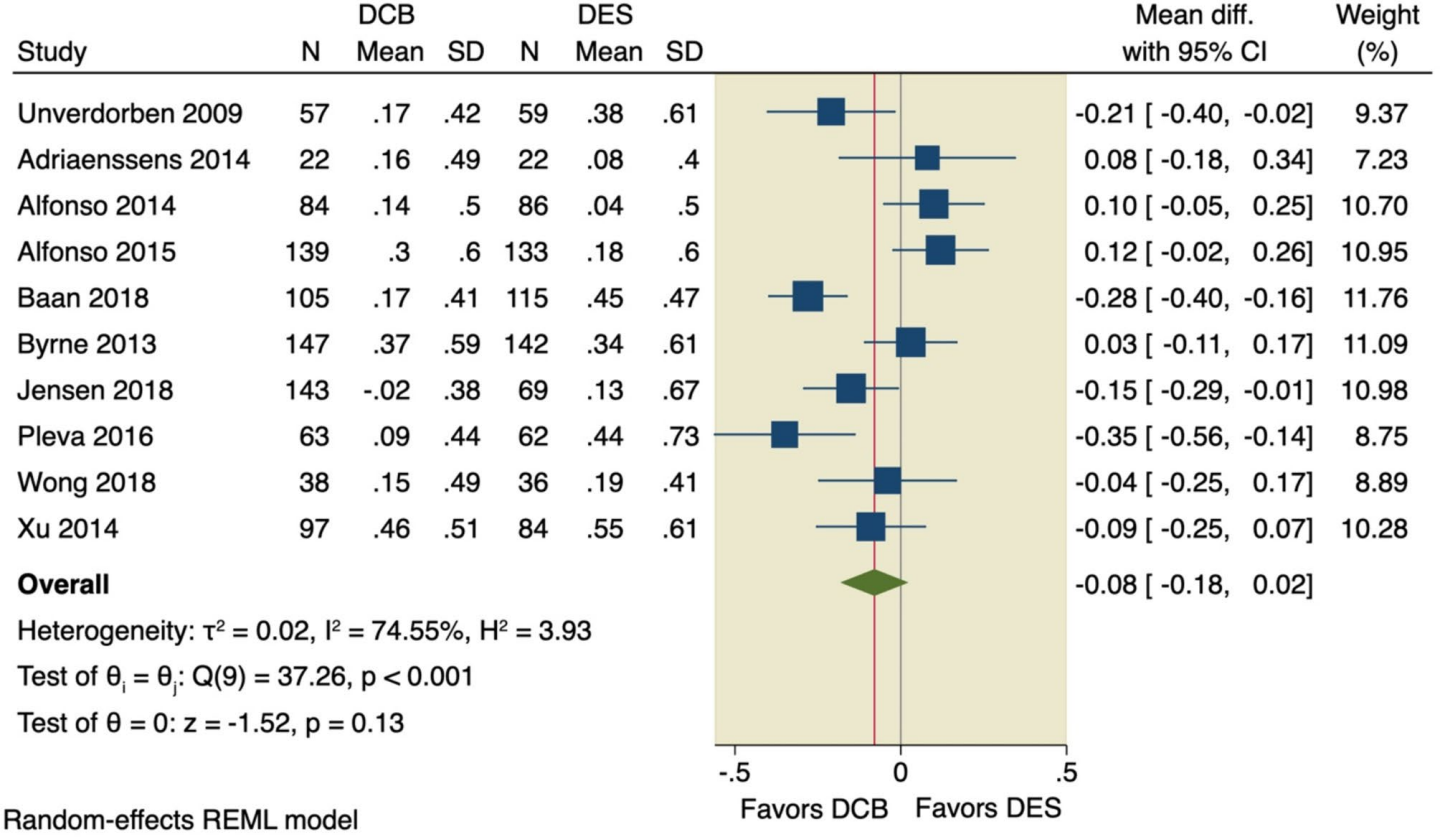
Pooled estimates from RCTs evaluating the effect of DCB on the LLL with a random-effects model. DCB: Drug-coated balloons; DES: drug-eluting stents; CI: confidence interval

**Fig. 8:**
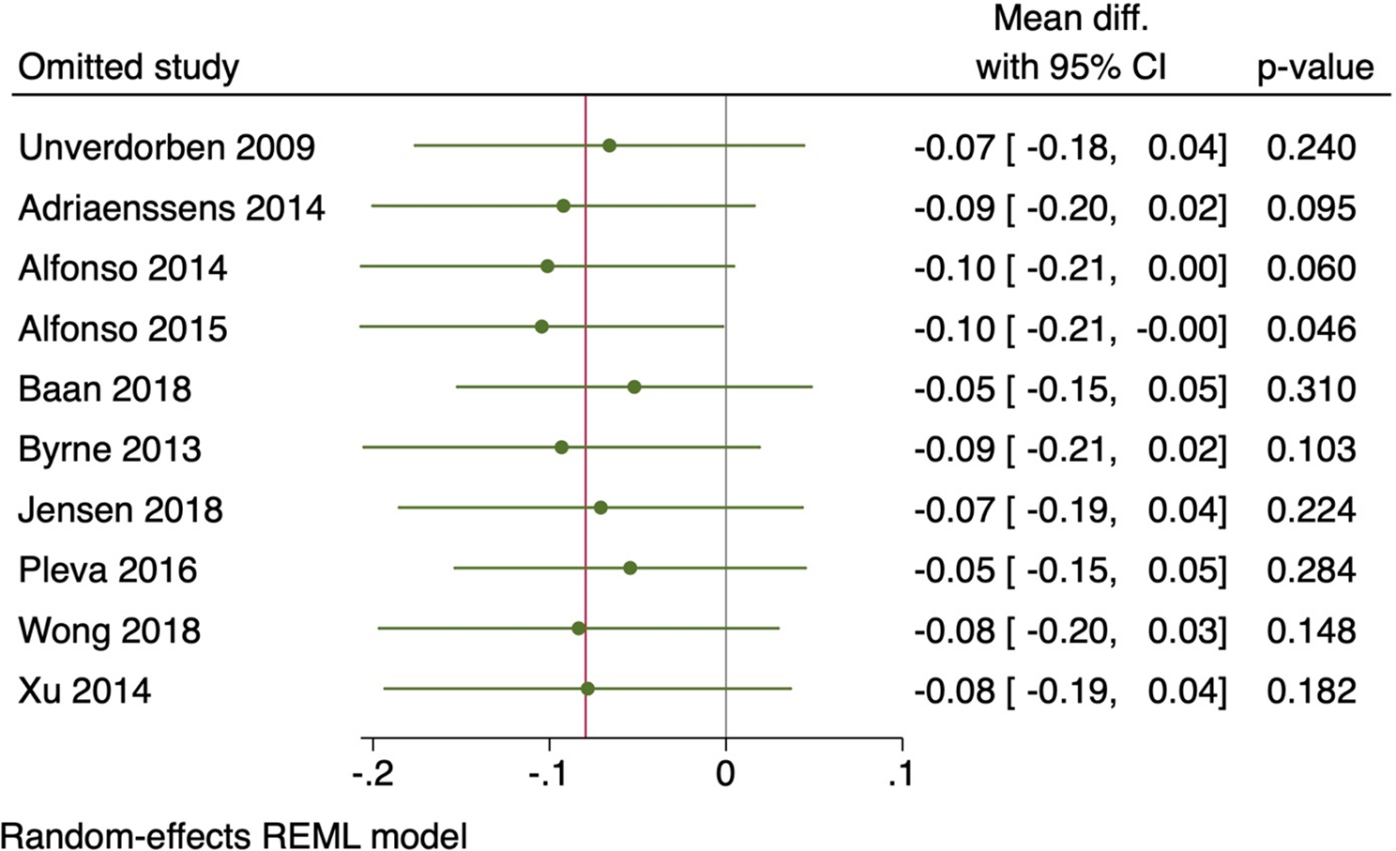
Leave-one-out analysis of LLL

LLL was assessed in all included studies, of which the pooled MD showed no significant difference between the DCB intervention and the DES intervention (MD = -0.08, 95% CI [-0.18, 0.02], *p* = 0.13); the pooled studies were significantly heterogenous (I^2^ = 74.55%, *p* < 0.001), as shown in Fig. 7. Leave-one-out model analysis was performed, and no single study had a disproportional effect on the overall MD, as shown in Fig. 8.

Four studies were inspected as outliers of the 95% CI of the precision area, using the Galbraith plot, indicating their heterogeneity from other studies; however, no other studies were computed in the funnel plot model to achieve stability, as shown in Supplementary Figs. S2–S3.

#### Secondary outcomes

Our analysis did not detect any significant difference between DCB intervention and DES intervention regarding in-stent binary restenosis (OR: 1.02, 95% CI [0.72 to 1.44], *p* = 0.92), all-cause death (OR = 0.79, 95% CI [0.52, 1.19], *p* = 0.26), cardiac death (OR = 0.83, 95% CI [0.47, 1.44], *p* = 0.50), the incidence of MI (OR = 0.89, 95% CI [0.6, 1.31], *p* = 0.54), the incidence of TVR (OR = 1.24, 95% CI [0.94, 1.63], *p* = 0.13), stent thrombosis (OR = 1.08, 95% CI [0.54, 2.16], *p* = 0.84), or MLD (MD = -0.08, 95% CI [-0.18, 0.02], *p* = 0.12), as shown in Supplementary Figs. S4–S10.

The pooled studies assessing all-cause death, cardiac death, MI, TVR, and stent thrombosis were homogenous with the following values, respectively: (I^2^ = 5.42%, *p* = 0.46; I^2^ = 0.00%, *p* = 0.95; and I^2^ = 0.00%, *p* = 0.97; I^2^ = 14.36%, *p* = 0.24; I^2^ = 0.00%, *p* = 0.97). However, the pooled studies assessing MLD were heterogenous (I^2^ = 60.6%, *p* = 0.01). Leave-one-out sensitivity analysis for MLD is shown in Supplementary Fig. S11.

On the other hand, DCB was associated with higher incidence of TLR compared to DES (OR: 1.54, 95% CI [1.2 to 1.99], *p* = 0.00), the pooled studies were homogenous (I^2^ = 0.00%, *p* = 0.26), as shown in Supplementary Fig. S12.

## Discussion

In this meta-analysis, we aimed to evaluate the efficacy of DCBs and DESs in patients with Coronary ISR. A total of 10 RCTs and six follow-up extensions were included in the analysis. Subgroup analyses were conducted based on intervention indications and follow-up periods to provide a more comprehensive assessment of the interventions’ effectiveness. In the short-term, DCBs showed better outcomes in reducing MACE than DESs, attributed to the absence of permanent foreign material reducing the risk of stent thrombosis and late re-stenosis [23]. DCBs also minimized the inflammatory response caused by DES implantation [24]. Previous studies without subgroup analysis reported no significant difference in MACE reduction between DCBs and DESs [25, 26]. However, DCBs were significantly more effective than DESs in reducing LLL and binary re-stenosis, such as all-cause death, cardiac death, myocardial infarction, target lesion revascularization, target vessel revascularization, stent thrombosis, and minimum lumen diameter. These latter findings were consistent with the previous meta-analyses [25–27].

ISR is a condition in which the artery narrows again after stent placement, leading to recurrent symptoms and potentially compromising the procedure’s outcomes. ISR is a common complication of PCI, with an incidence ranging from 5 to 50%, depending on the patient and procedural factors and the type of stent used [28, 29]. The pathophysiology of ISR is complex and multifactorial, involving inflammation, neointimal hyperplasia, and other factors contributing to tissue overgrowth within the stent [30]. Therefore, ISR prevention and management remain a significant challenge in clinical practice, and several strategies, including DES, non-invasive imaging, and pharmacotherapy, have been proposed to reduce the incidence and improve the outcomes of this condition [31, 32].

A considerable literature, comprising both observational studies and randomized trials, has been carried out to explore the efficacy of DCB and DES in addressing re-stenosis in both BMS and DES [6, 33–35]. However, few randomized clinical trials have been published that directly compare DCB with current-generation DES [14, 15]. These trials consistently employed a comparative approach, evaluating the SeQuent Please paclitaxel-eluting balloon (DEB) against the XIENCE everolimus-eluting stent (EES), which is widely regarded as the reference device within its category. The XIENCE EES has undergone extensive investigation as a DES, while the SeQuent Please paclitaxel-eluting balloon has been extensively studied as a DCB [36–38]. The Alfonso et al. trial (2015), comprising 189 patients, demonstrated that using EES led to superior late angiographic outcomes, as evidenced by a larger MLD at the 9-month follow-up compared to DCB [11]. In the study by Baan et al., the requirement for TVR was found to be statistically comparable between the two groups, but there was a numerical tendency for a higher incidence in the DCB arm [4]. Conversely, the study by Pleva et al., which included 136 patients, reported more favorable angiographic outcomes with DCB than EES, indicated by a smaller late lumen loss at the 12-month follow-up [16].

The Drug-eluting stent and drug-coated balloon AngiopLasty for the occUrrence of coronary in-Stent restenosis (DAEDALUS) study was a comprehensive meta-analysis that compared the effectiveness of DES and DCB angioplasty in treating coronary ISR. The study included individual patient data from 10 randomized clinical trials, with a total of 1,976 participants. The results showed that at 3 years, DCB angioplasty was associated with a moderately higher risk of target lesion revascularization compared to repeat DES implantation, with a significant interaction between treatment effect and the type of restenosed stent. The safety endpoint of all-cause death, myocardial infarction, or target lesion thrombosis was similar between the two groups. However, a subgroup analysis suggested a lower incidence of adverse events with paclitaxel-coated balloons compared to first-generation DES. The study also found that DCB and DES had comparable efficacy and safety profiles in treating bare-metal stent ISR, while repeat DES implantation was more effective in treating DES-ISR, albeit with a numerical excess in the composite safety endpoint. The different efficacy of DCB between BMS-ISR and DES-ISR may be due to variations in individual susceptibility to antiproliferative drug-mediated suppression and the occurrence of neo atherosclerosis [39].

Several meta-analyses also have been published comparing different treatments for ISR. In a study by Lee et al., it was found that both paclitaxel-coated balloons and DES were associated with a significant reduction in TLR and MACE compared to plain old balloon angioplasty (POBA). The OR for TLR was 0.28, and for MACE was 0.84 when comparing DCB or DES to POBA [40]. However, there was no significant difference between DCB and DES groups. Similarly, in a study by Mamuti et al., no significant difference in MACE was observed in the treatment of BMS or DES-ISR when comparing DCB to paclitaxel-eluting stents (PES) or EES. The RR for MACE was 1.04, indicating no significant difference between DCB and PES/EES (*P* = 0.80) [41]. These meta-analyses provide evidence that DCB and DES are effective in reducing TLR and MACE compared to POBA, while there is no significant difference between DCB and DES regarding their efficacy.

Additionally, DCB has shown comparable effectiveness to PES/EES in the treatment of BMS/DES-ISR, and both DES and DCB appear to be superior to POBA in reducing the risk of TLR and TVR in DES-ISR However, previous meta-analyses have major drawbacks: Geo et al. included only four RCTs, most of their studies were single-arm studies, and Maumti et al. included only four trials. Additionally, many studies have been conducted after these meta-analyses [4, 9, 10, 21, 22].

### Multilayer ISR

The management of multi-layered ISR presents a compelling and often challenging clinical scenario. To address this, a non-randomized study examined the outcomes of recurrent ISR lesions treated with at least three prior stents, comparing the efficacy of drug-coated balloons DCBs and second-generation DES. The study analyzed 171 lesions, with 82 lesions treated with second-generation DES and the remaining lesions treated with DCBs. Upon two-year follow-up, the study revealed no significant difference in outcomes between the DES and DCB treatment groups. However, it is noteworthy that the DCB group exhibited a noticeably higher rate of major adverse cardiac events (43.5% vs. 28.8%; *P* = 0.21) [42]. This finding underscores the importance of careful patient selection and consideration of various factors when deciding between DES and DCB for the treatment of complex ISR lesions. While DCB offer an elegant solution, their long-term efficacy in patients with three or more layers is unsatisfactory [43]. Alternative “leave-nothing-behind” approaches have not demonstrated superiority over DCB in this complex patient population [44]. Further research is imperative to break this therapeutic deadlock. In carefully selected patients with recalcitrant ISR and extensive areas of myocardium at risk, coronary surgery should be regarded not as a concession or failure but as the most prudent strategy to prevent recurrent restenosis and its adverse consequences.

The Tokyo Registry study analyzed 304 patients with 333 ISR lesions who underwent treatment with DEB. The patients were categorized into three groups based on the number of previous metal stent layers: 166 (54%) with one stent layer, 87 (29%) with two layers, and 51 (17%) with three or more layers. The study found that the one-year MACE rate was significantly higher in the group with three or more metal layers (43.1%) compared to the groups with fewer layers [45].

### Imaging role

The utilization of coronary physiology and intravascular imaging as adjunctive tools during percutaneous coronary interventions has been extensively studied, with a preponderance of evidence signifying enhanced outcomes when compared to sole angiographic guidance. Although limited in scope, existing reports examining the efficacy of these techniques in conjunction with drug-coated balloon interventions do not diverge in their conclusions from those observed with drug-eluting stents. Fractional flow reserve and pressure gradient measurements, as examples of physiological metrics, have been validated in the context of drug-coated balloon interventions, as evidenced by extant data [46].

A study employing serial optical coherence tomography revealed intriguing morphologic transformations within lesions undergoing ISR treatment with DCB angioplasty [47]. Notably, postprocedural dissections were observed specifically in segments where ISR presented with greater severity [47]. These dissections, elusive to angiographic imaging, were left untreated, yet exhibited complete resolution upon invasive follow-up examination. The integration of intravascular imaging modalities, such as intravascular ultrasound and optical coherence tomography, offers valuable insights into the underlying mechanisms of ISR [48]. By elucidating lesion-specific mechanical and histopathological factors, these imaging techniques inform the decision-making process between DCB and DES interventions, as well as the necessity for lesion preparation strategies prior to DCB application, including aggressive predilation with specialized balloons and high-pressure inflation [48].

Our study’s strengths are noteworthy, as it represents a comprehensive analysis of 16 RCTs with a total sample size exceeding 2,500 patients. Subgroup analyses were conducted based on follow-up duration and an indication of the intervention, providing a comprehensive understanding of the effectiveness of DCBs compared to other treatments. Additionally, the study employed various statistical methods, such as funnel plots, leave-one-out analysis, and Galbraith plots, to detect publication bias and outliers, thereby improving the robustness of the findings. Also, we ran a TSA analysis to test the robustness of the evidence pooled from the included studies which was not applied in the past meta-analyses.

The study’s limitations encompass several aspects. First, significant heterogeneity was observed in certain pooled analyses, which can be attributed to clinical heterogeneity stemming from the pooling of different generations of DES with DCB. Additionally, there was a lack of stratification of patients based on the number of metal-layered DES-ISR, methodological differences arising from variations in patient populations. Moreover, some of the studies included in the analysis had small sample sizes, potentially reducing the statistical power to detect significant differences between interventions. Lastly, despite the rigorous inclusion of only RCTs to ensure the highest level of evidence, the quality of the study’s findings may still be influenced by the potential risk of bias inherent in the individual studies incorporated in the analysis.

## Conclusion

Our meta-analysis provides compelling evidence for the superior efficacy of DCBs over DES in treating coronary ISR. The analysis included six follow-up extensions, demonstrating that DCBs reduce MACE in the short term due to reduced stent thrombosis and late restenosis. DCBs also minimize inflammatory responses associated with DES. These findings directly impact daily practice, especially with the prevalent use of DES in ISR treatment, highlighting DCBs as the possible preferred intervention for improved ISR outcomes. Furthermore, to substantiate these findings, RCTs with a larger sample size and extended follow-up period is warranted to enhance the robustness of the current results.

### Daily practice summary

The European Society of Cardiology’s guidelines on myocardial revascularization advocate the utilization of DCBs specifically for the treatment of ISR, with no extensions to other indications [49]. This cautious recommendation reflects the state of clinical knowledge at the time of guideline development and stands in contrast to the broader contemporary usage of DCBs in Europe. However, in recent years, there has been a significant expansion of data and clinical trials evaluating the efficacy of DCBs in treating de novo coronary lesions. The treatment paradigm for de novo lesions with DCBs offers a unique advantage by circumventing the need for permanent implants, thereby reducing the long-term risks associated with such devices [50]. The success of DCB angioplasty hinges on meticulous lesion preparation, and in cases of diffuse coronary artery disease, it can serve as a valuable adjunct to DES, minimizing the number and length of stents required. This hybrid approach underscores the importance of proper training and education for interventional cardiologists to ensure its effective implementation. Despite the accumulating clinical data and experience with DCBs in Europe, their usage for coronary artery disease treatment has not been approved by the United States Food and Drug Administration, warranting the need for large-scale randomized clinical trials powered for clinical endpoints to bridge this regulatory gap.

## Electronic supplementary material

Supplementary Material 1

## Data Availability

All data generated during this study are illustrated in this article and in supplementary data.

## Abbreviations

DCB: Drug-coated balloons
DES: Drug-eluting stents
ISR: In-stent restenosis
CAD: Coronary artery disease
PCI: Percutaneous coronary intervention
PRISMA: Systematic Reviews and Meta-analysis
TSA: Trial sequential analysis
OR: Odds ratio
NA: Not Assigned
