# Supplementary material for: Drug-coated balloons versus drug-eluting stents in patients with in-stent restenosis: An updated meta-analysis with trial sequential analysis
Source: J Cardiothorac Surg. 2024 Nov 6;19:624. doi: 10.1186/s13019-024-03046-6 (PMC11539716; doi:10.1186/s13019-024-03046-6)
Supplement: Supplementary file 1 — Supplementary Material 1 [file 13019_2024_3046_MOESM1_ESM.docx]

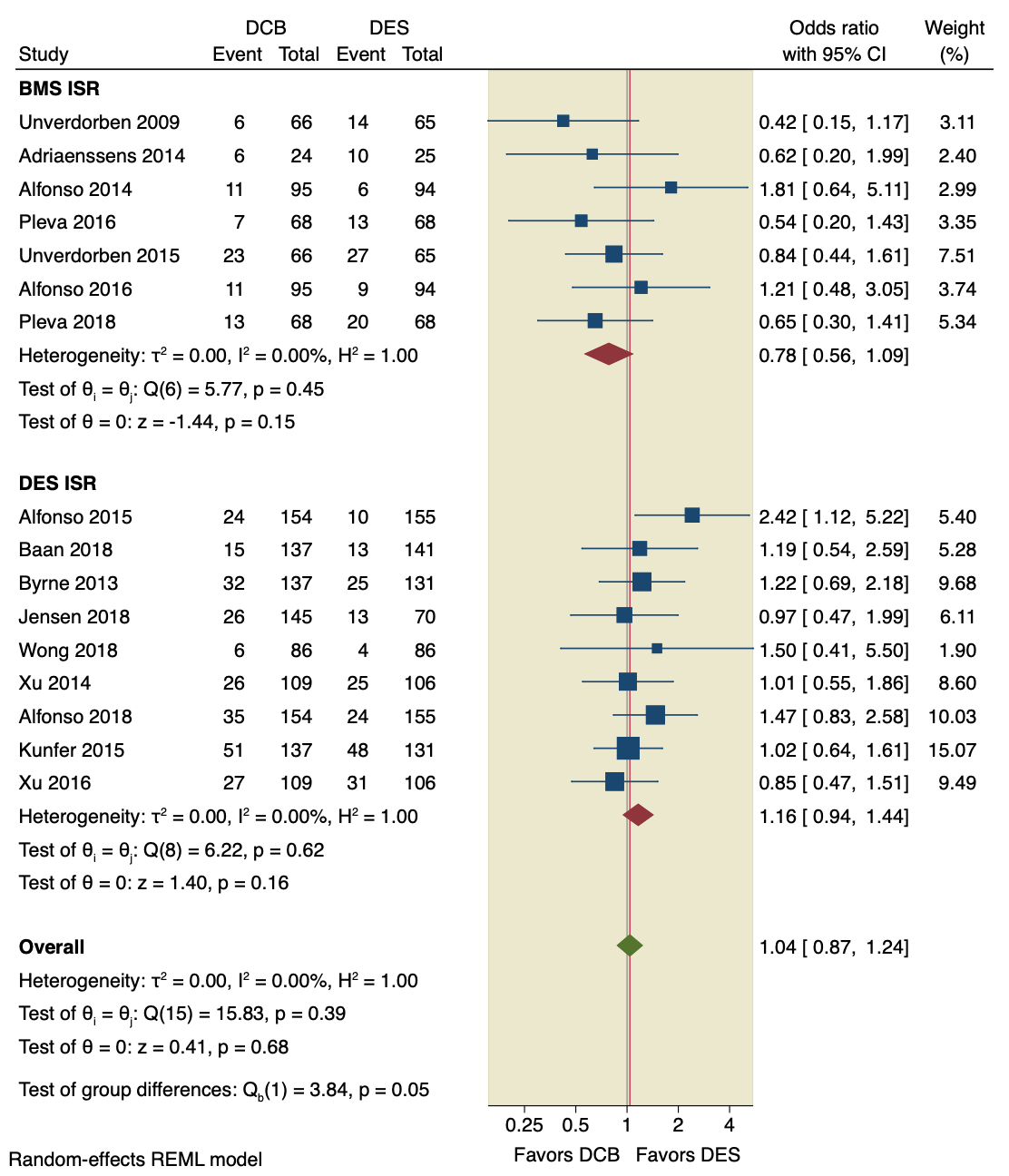


**Supplementary Figure 1:** Pooled estimates from RCTs evaluating the effect of DCB on the incidence of MACE with a random-effects model according to the intervention used. DCB: Drug-coated balloons; DES: drug-eluting stents; CI: confidence interval.


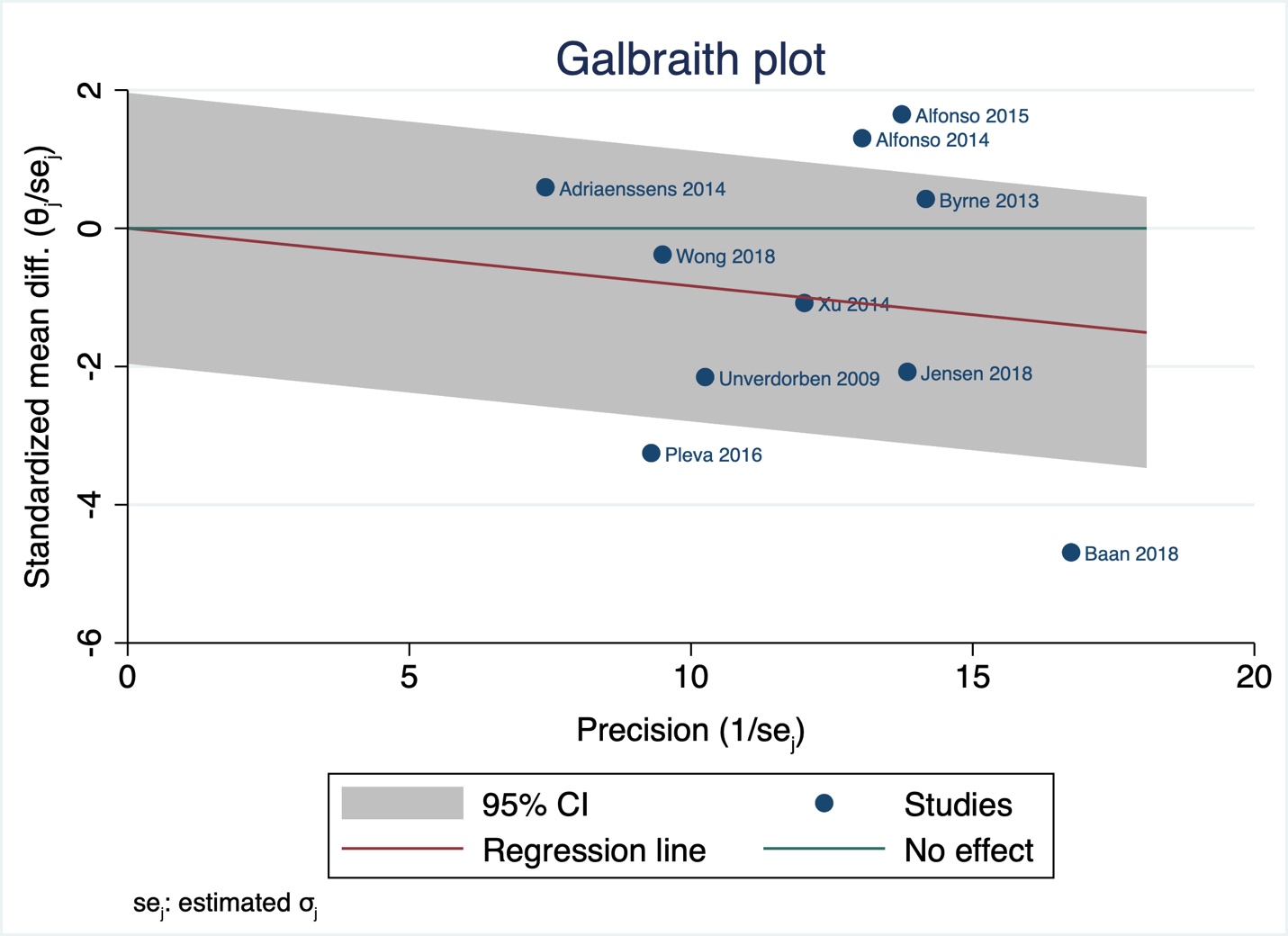


**Supplementary Figure 2:** Galbraith plot of LLL.


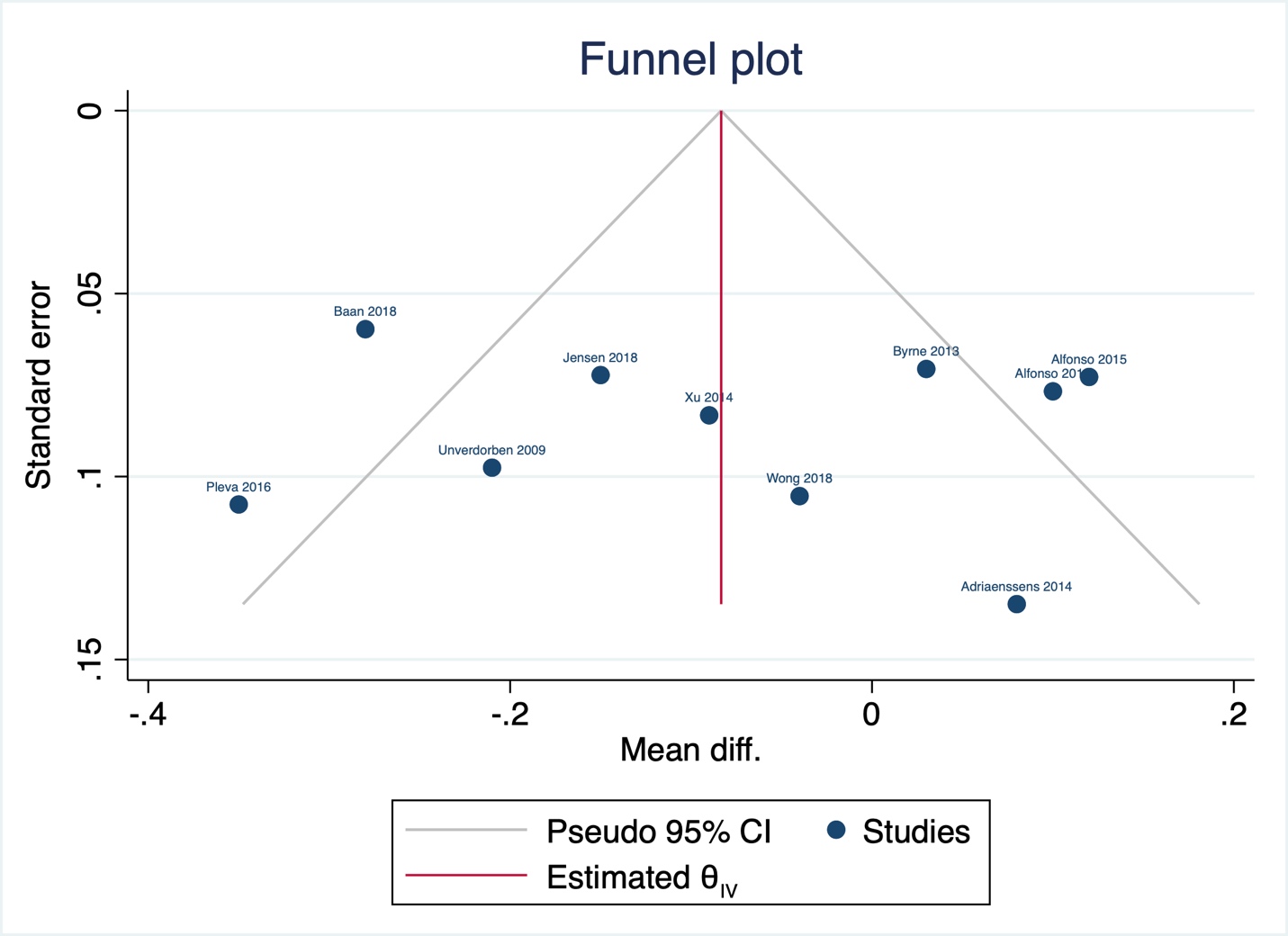


**Supplementary Figure 3:** Funnel plot of LLL.


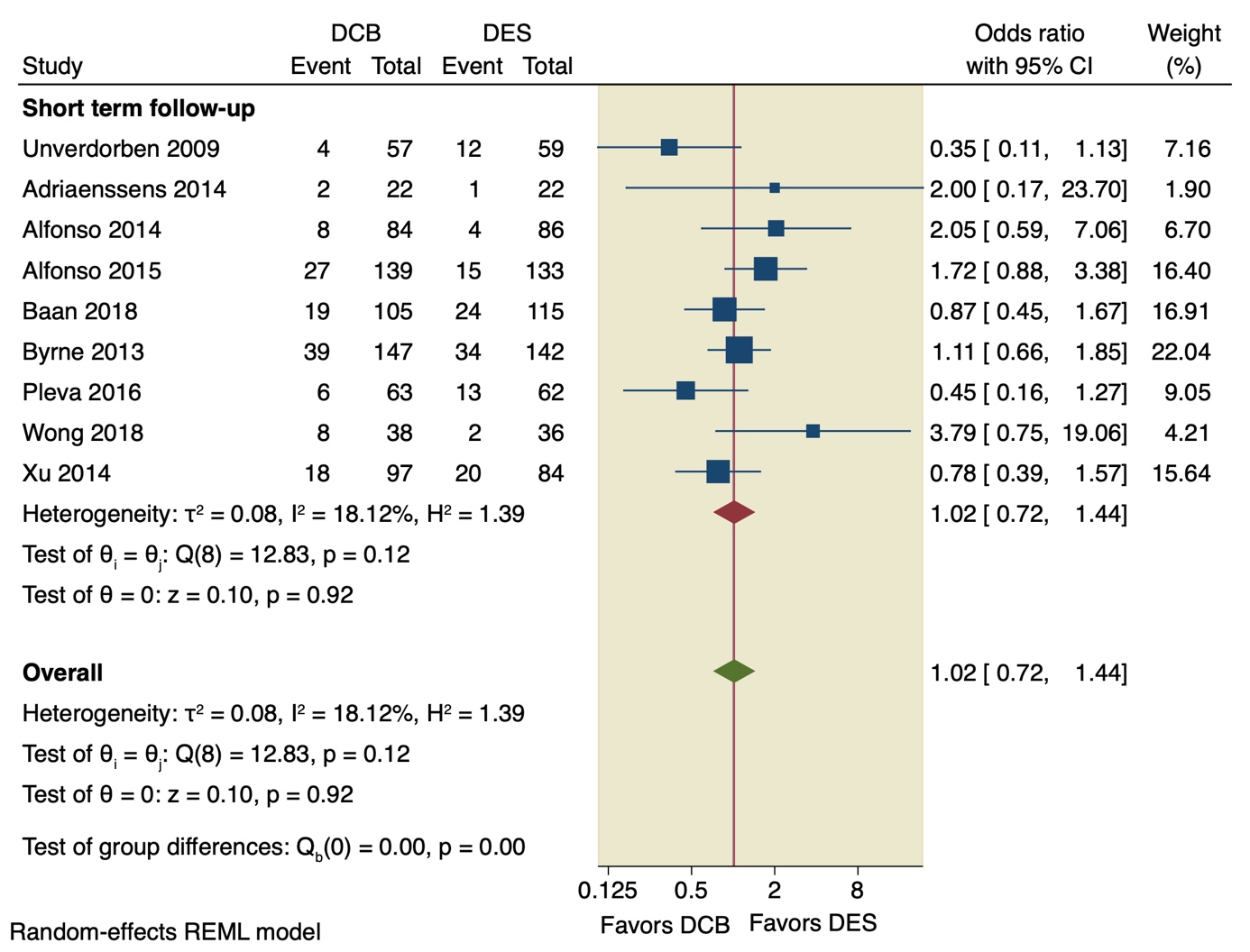


**Supplementary Figure 4:** Pooled estimates from RCTs evaluating the effect of DCB on the incidence of in-stent binary restenosis with a random-effects model. DCB: Drug-coated balloons; DES: drug-eluting stents; CI: confidence interval.


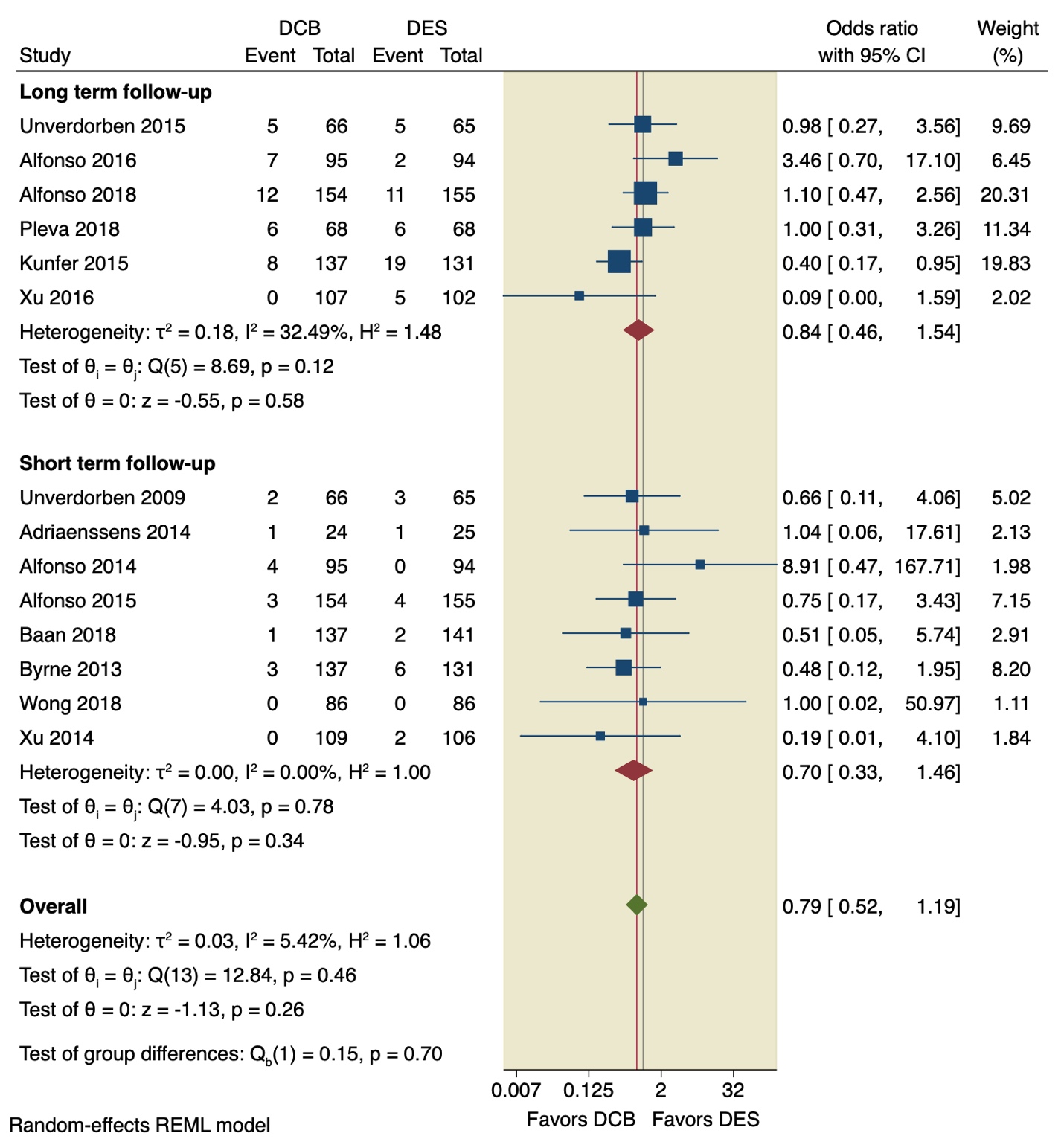


**Supplementary Figure 5:** Pooled estimates from RCTs evaluating the effect of DCB on the incidence of all-cause death with a random-effects model. DCB: Drug-coated balloons; DES: drug-eluting stents; CI: confidence interval.


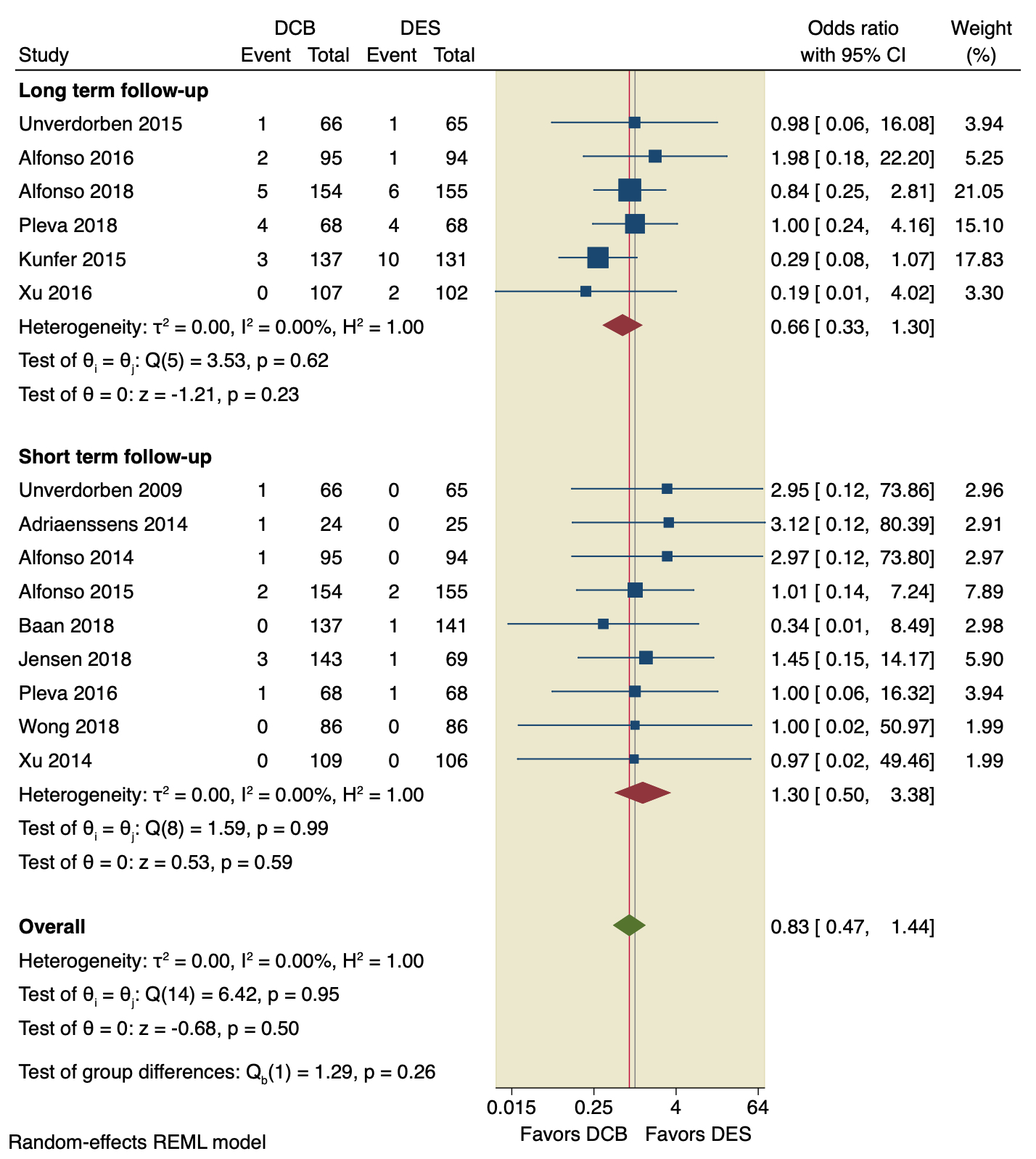


**Supplementary Figure 6:** Pooled estimates from RCTs evaluating the effect of DCB on the incidence of cardiac death with a random-effects model. DCB: Drug-coated balloons; DES: drug-eluting stents; CI: confidence interval.


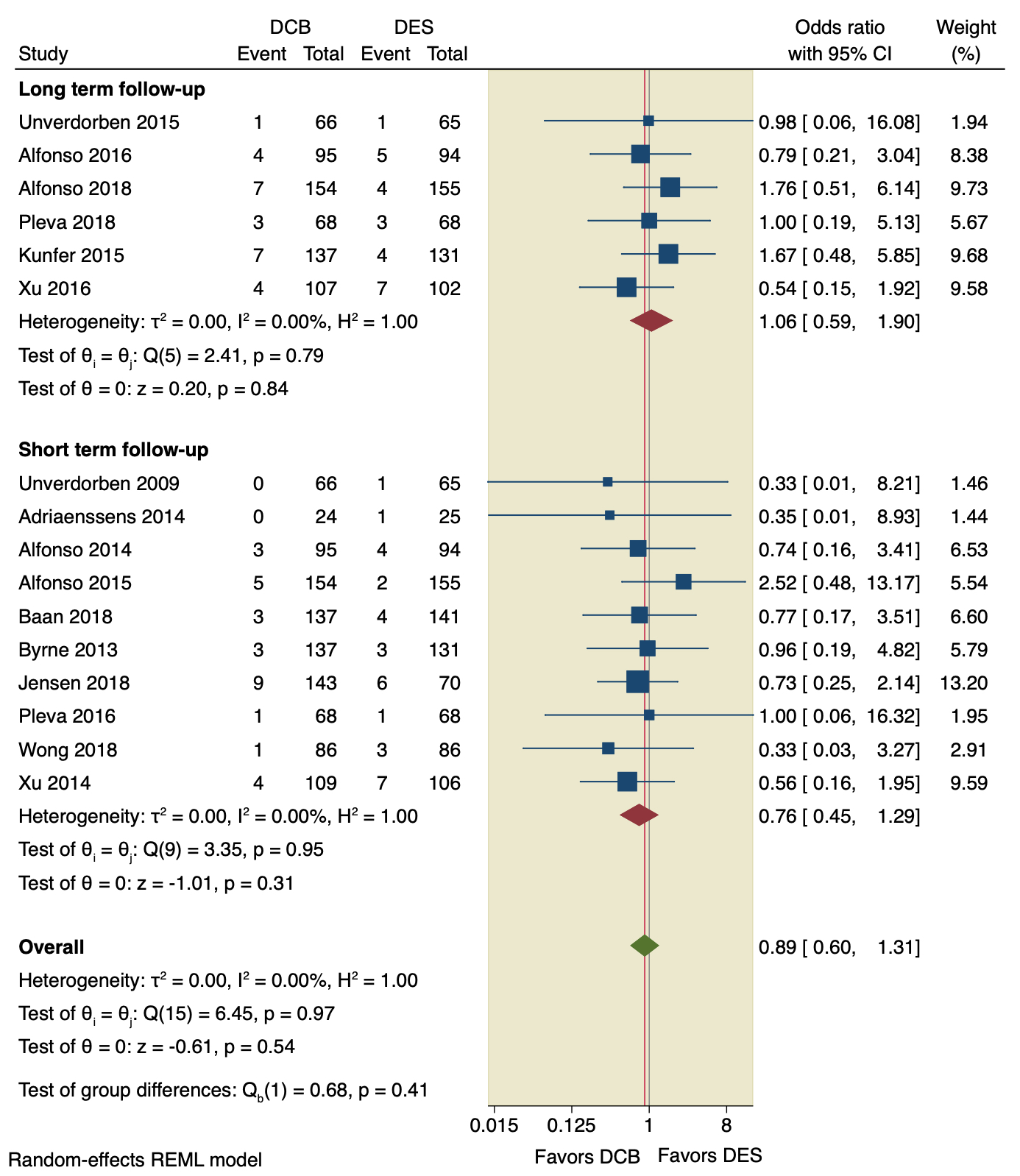


**Supplementary Figure 7:** Pooled estimates from RCTs evaluating the effect of DCB on the incidence of MI with a random-effects model. DCB: Drug-coated balloons; DES: drug-eluting stents; CI: confidence interval.


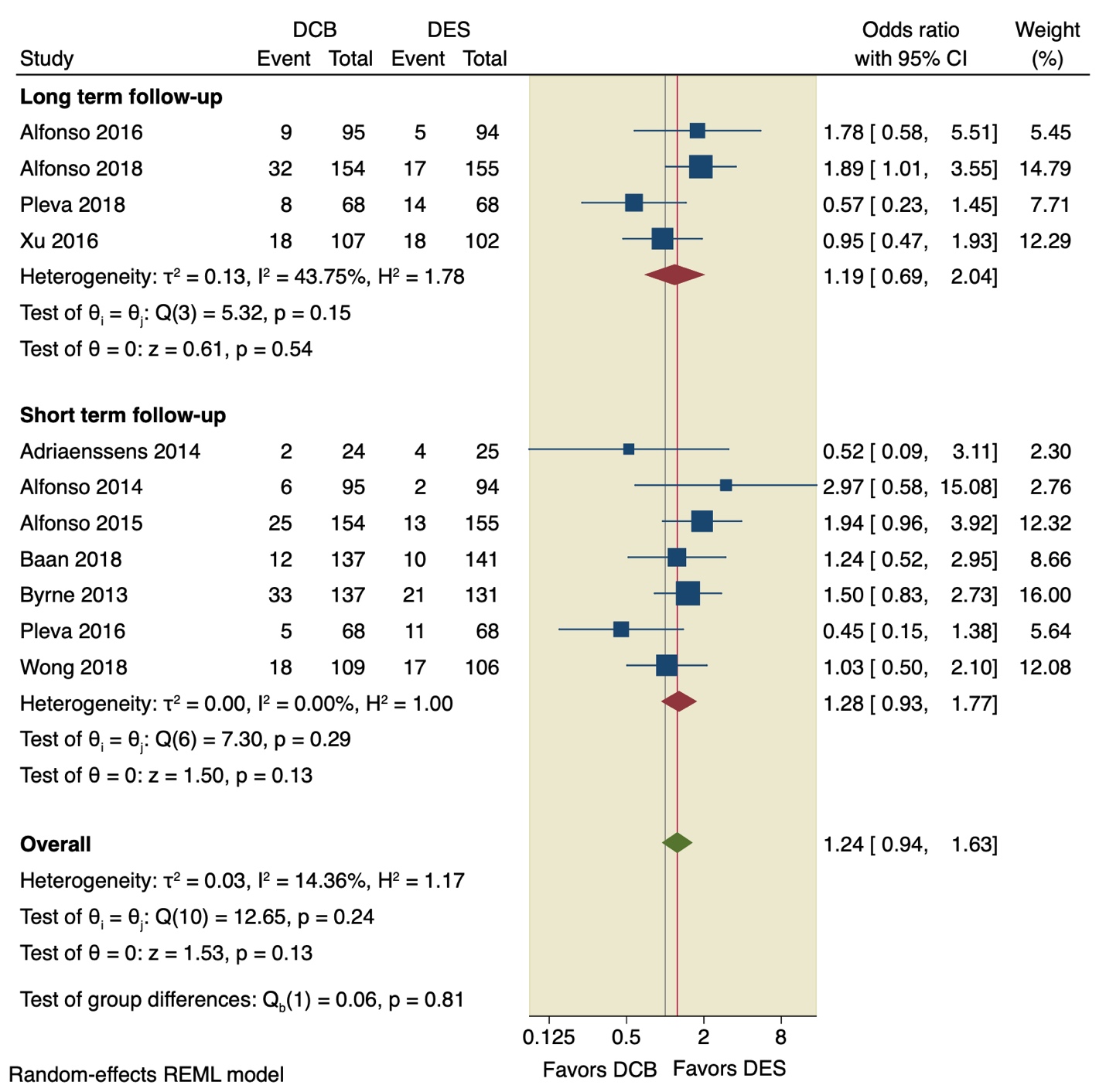


**Supplementary Figure 8:** Pooled estimates from RCTs evaluating the effect of DCB on the incidence of TVR with a random-effects model. DCB: Drug-coated balloons; DES: drug-eluting stents; CI: confidence interval.


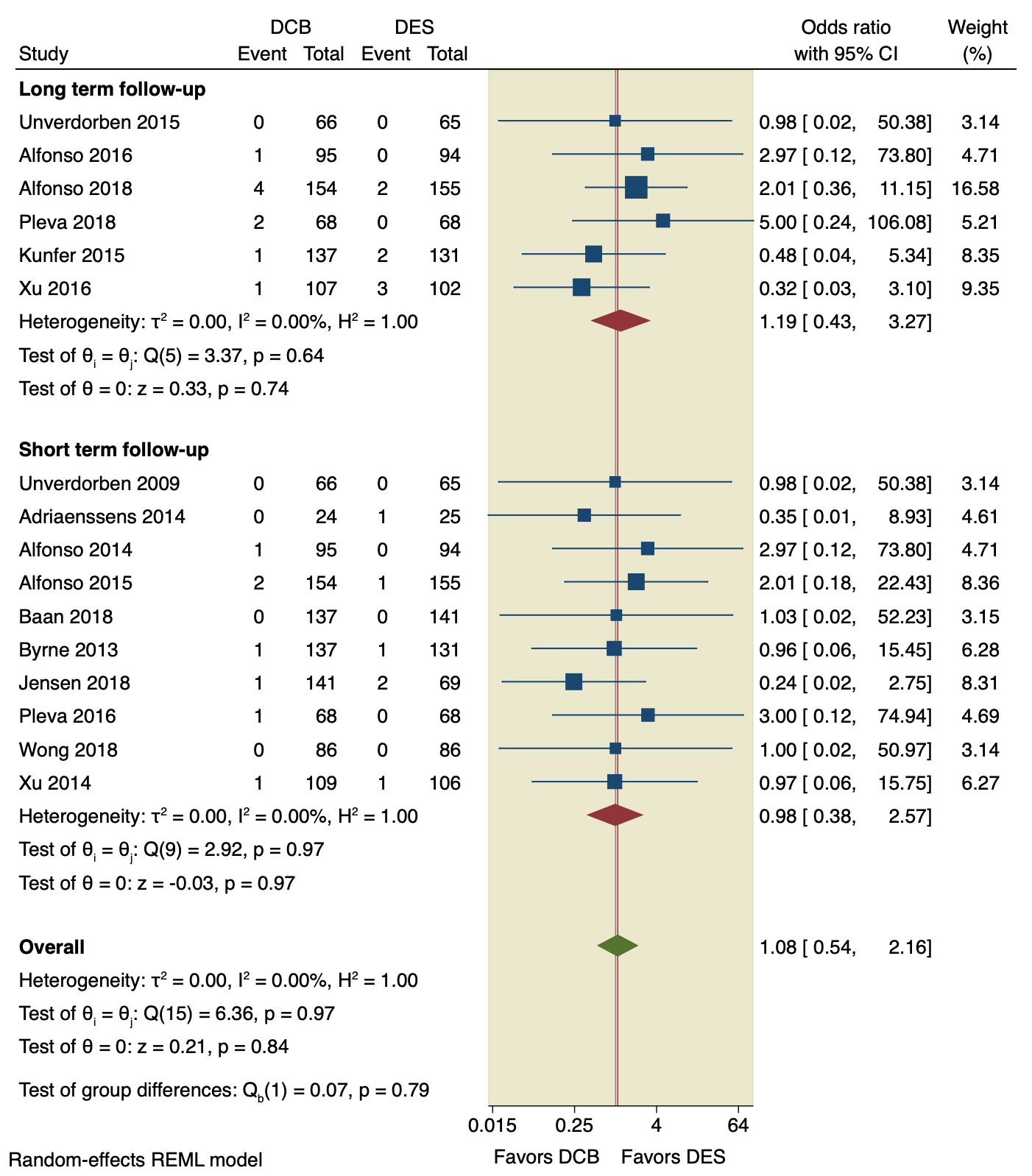


**Supplementary Figure 9:** Pooled estimates from RCTs evaluating the effect of DCB on the incidence of stent thrombosis with a random-effects model. DCB: Drug-coated balloons; DES: drug-eluting stents; CI: confidence interval.


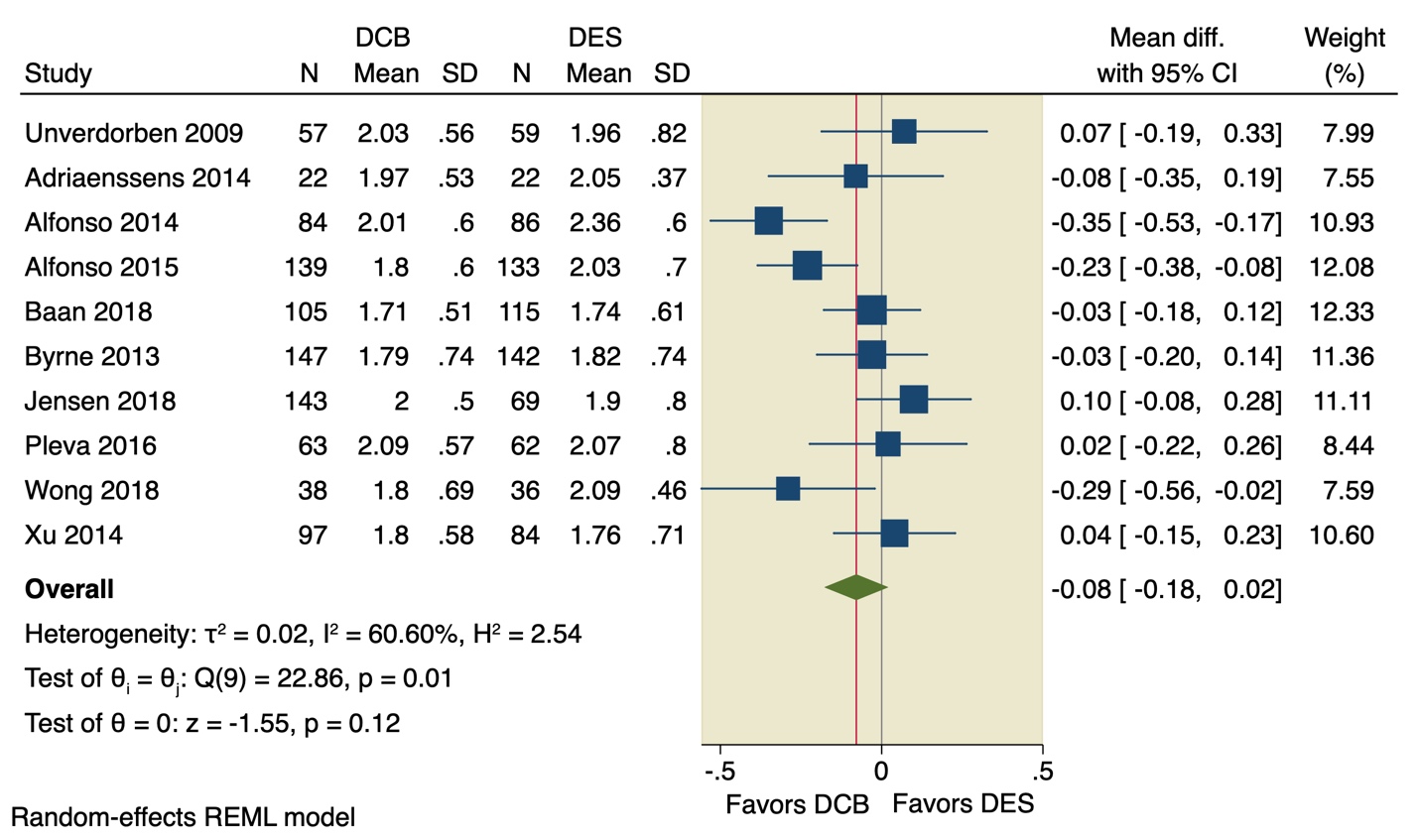


**Supplementary Figure 10:** Pooled estimates from RCTs evaluating the effect of DCB on the MLD with a random-effects model. DCB: Drug-coated balloons; DES: drug-eluting stents; CI: confidence interval.


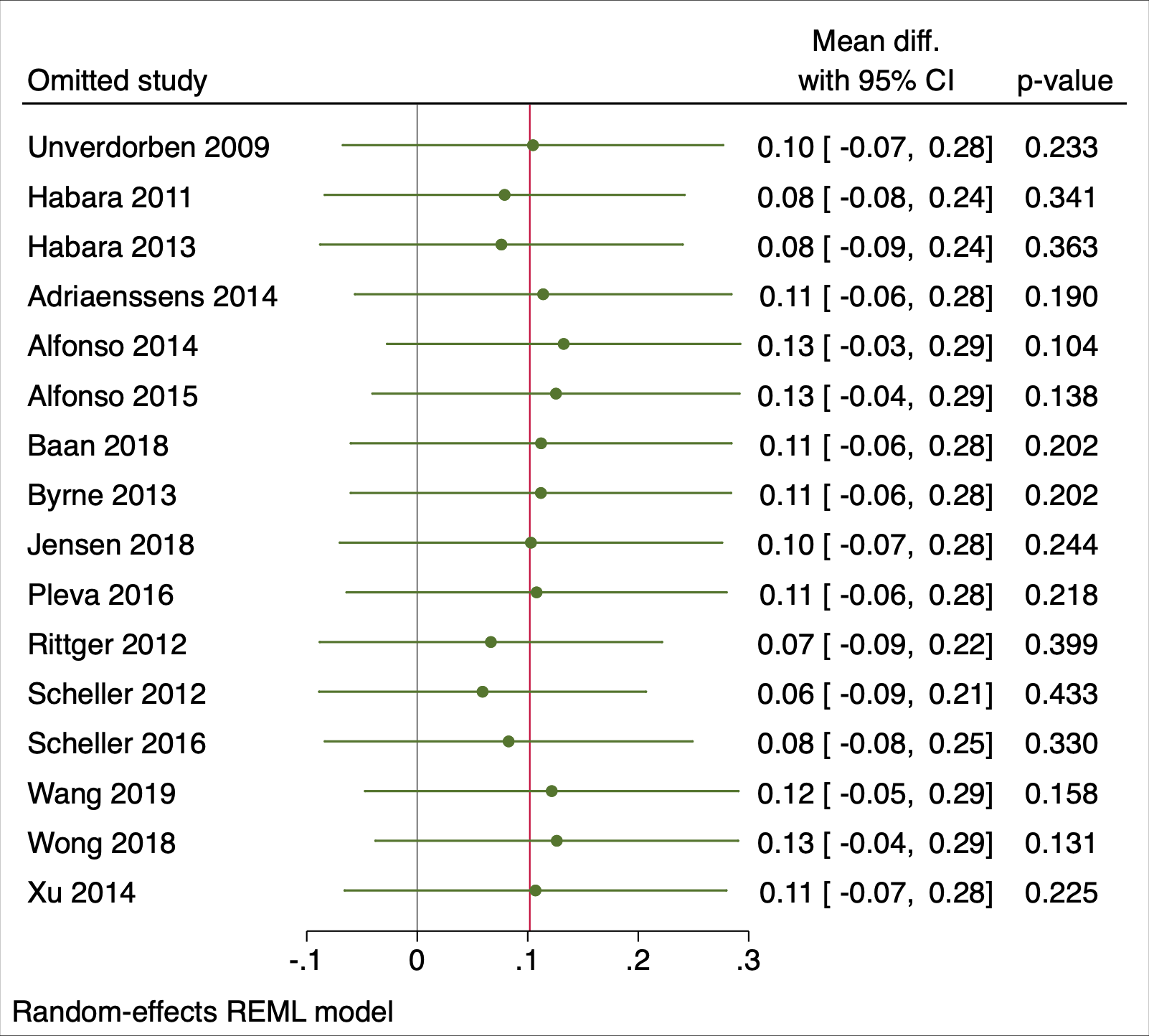


**Supplementary Figure 11:** Leave-one-out of MLD.


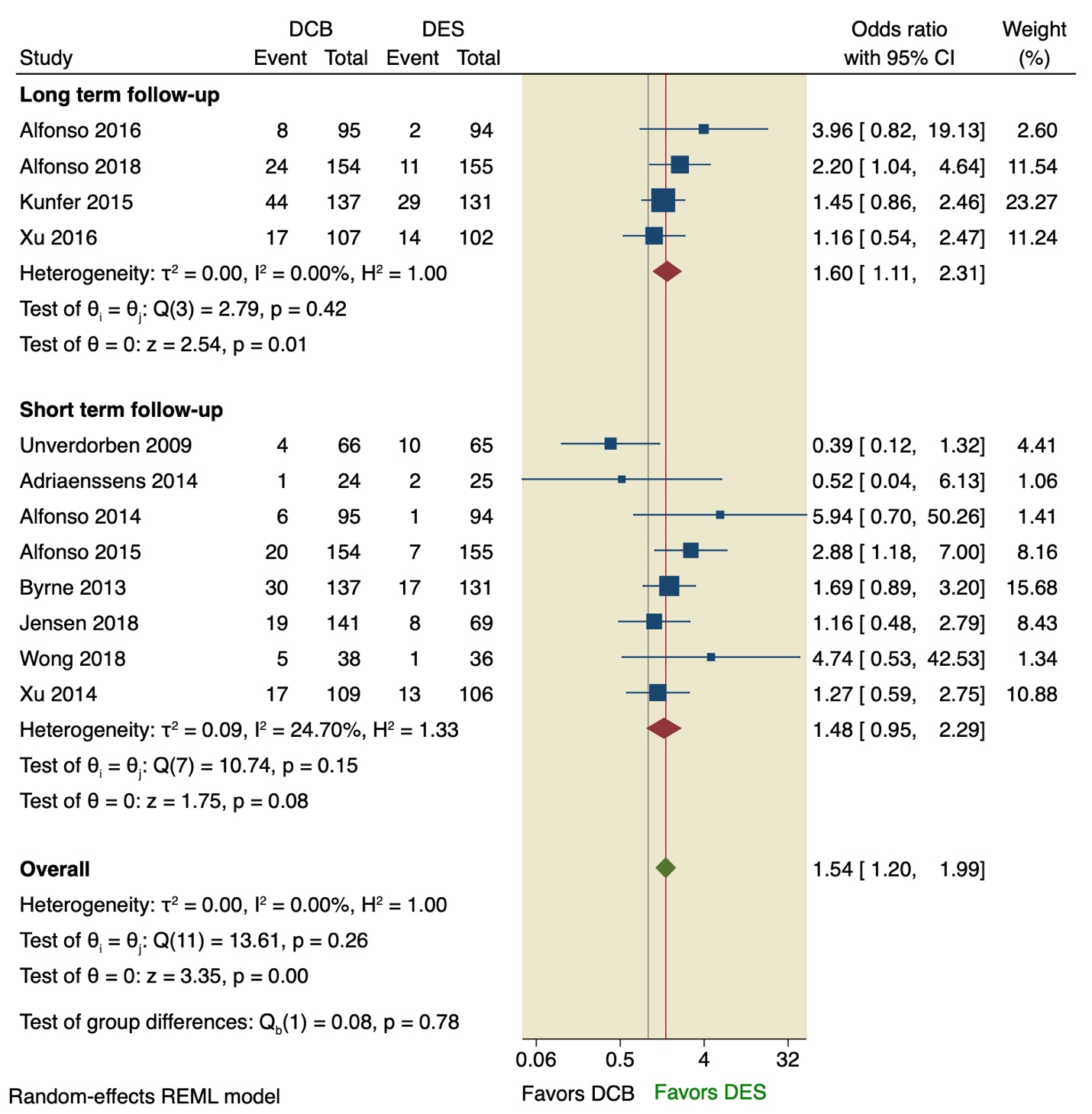


**Supplementary Figure 12:** Pooled estimates from RCTs evaluating the effect of DCB on the TLR with a random-effects model. DCB: Drug-coated balloons; DES: drug-eluting stents; CI: confidence interval.
